# Microbial Biofilm Decontamination on Dental Implant Surfaces: A Mini Review

**DOI:** 10.3389/fcimb.2021.736186

**Published:** 2021-10-08

**Authors:** Jagjit Singh Dhaliwal, Nurul Adhwa Abd Rahman, Long Chiau Ming, Sachinjeet Kaur Sodhi Dhaliwal, Joe Knights, Rubens Ferreira Albuquerque Junior

**Affiliations:** ^1^ Pengiran Anak Puteri Rashidah Sa'adatul Bolkiah Institute of Health Sciences, Universiti Brunei, Darussalam, Gadong, Brunei; ^2^ Faculdade de Odontologia, Universidade de São Paulo, Butantã, Brazil

**Keywords:** dental implant, decontamination, titanium, bacteria, biofilm

## Abstract

**Introduction:**

After insertion into the bone, implants osseointegrate, which is required for their long-term success. However, inflammation and infection around the implants may lead to implant failure leading to peri-implantitis and loss of supporting bone, which may eventually lead to failure of implant. Surface chemistry of the implant and lack of cleanliness on the part of the patient are related to peri-implantitis. The only way to get rid of this infection is decontamination of dental implants.

**Objective:**

This systematic review intended to study decontamination of microbial biofilm methods on titanium implant surfaces used in dentistry.

**Methods:**

The electronic databases Springer Link, Science Direct, and PubMed were explored from their inception until December 2020 to identify relevant studies. Studies included had to evaluate the efficiency of new strategies either to prevent formation of biofilm or to treat matured biofilm on dental implant surfaces.

**Results and Discussion:**

In this systematic review, 17 different groups of decontamination methods were summarized from 116 studies. The decontamination methods included coating materials, mechanical cleaning, laser treatment, photodynamic therapy, air polishing, anodizing treatment, radiation, sonication, thermal treatment, ultrasound treatment, chemical treatment, electrochemical treatment, antimicrobial drugs, argon treatment, and probiotics.

**Conclusion:**

The findings suggest that most of the decontamination methods were effective in preventing the formation of biofilm and in decontaminating established biofilm on dental implants. This narrative review provides a summary of methods for future research in the development of new dental implants and decontamination techniques.

## Introduction

Dental implants are number one choice for replacement of teeth. It is a valuable option in several treatment scenarios, e.g., (i) when teeth are lost to non-periodontal diseases, infection due to caries, traumatic injuries; (ii) loss of teeth due to periodontal diseases but the remaining teeth can be maintained; and (iii) combined scenarios requiring replacement of missing teeth (Greenwell et al., 2019). An implant is typically composed of titanium or titanium alloys because of its biocompatibility and mechanical properties. Recent studies show that titanium dental implants survived for more than 20 years, regardless of their long-term exposure to the oral environment (Chen et al., 2017; Albrektsson et al., 2019). After insertion into the bone, implant osseointegration is expected to occur and is fundamental for its long-term success (Alghamdi, 2018). However, dental implants cannot be used for treatment of periodontal or dental disease (Greenwell et al., 2019). In addition, inflammatory reaction and infection around the implants may occur and can lead to implant failure, a phenomenon called peri-implantitis (Berglundh et al., 2019). Peri-implantitis is caused by plaque bacteria, leading to inflammation of mucosa and subsequent bone loss surrounding the implant, which may ultimately result in total implant failure (Berglundh et al., 2019). Surface chemistry and lack of cleanliness are intimately associated with peri-implantitis (Rupp et al., 2018).

Implant loss often triggers a devastating psychological impact on patients’ lives, with significantly high financial losses to families and healthcare systems (Alzahrani and Gibson, 2018; Frazadmoghadam et al., 2018; Hoeksema et al., 2018). Peri-implantitis is reported to occur at different levels of severity, with about 10% of them being lost within a 5-year period (Koldsland et al., 2010; Caton J. et al., 2018). It has been reported that peri‐implantitis takes place in 3–47% of dental implants (Koldsland et al., 2010; Matarazzo et al., 2018; French et al., 2019). Peri-implantitis is frequently related to periodontal pathogens and usually treated with antibiotics (Ting et al., 2018; Hussain et al., 2018). Bacterial biofilm has been advocated to be the leading etiological factor in the causation of peri-implantitis. Biofilm is a highly structured, matrix-enclosed bacterial community in a sessile state (Magana et al., 2018). Biofilm formation is a typical feature of bacterial growth, causing evasion from host cells and avoiding competition with other microbial communities (Guilhen et al., 2017). Several steps are needed to form biofilms, which include (i) planktonic bacteria adhesion on the surface, (ii) adhesion on the surface to establish a firm platform, (iii) co-aggregation with other bacteria, which further stabilizes their buildup architecture, (iv) growth by absorbing nutrition from the environment until maturation phase, and (v) detachment of a portion of biofilms to invade another vulnerable site (Guilhen et al., 2017). It is more challenging to eradicate established biofilm colonization than to eradicate the circulating contamination itself (*i.e.*, planktonic microbiota). This significantly increases resistance to antibiotic treatment (Arciola et al., 2018). Resident microbiota and matrix in biofilm may hinder access of antimicrobials locally, preventing their penetration into deeper layers of biofilm (Kuang et al., 2018). Therefore, it is deemed imperative that studies in the future focus on the prevention or reduction of biofilm formation.

Several techniques for decontamination of implants have been used over the years. However, none of them have successfully produced optimal results. Mechanisms underpinning these processes are still not understood fully and decontamination of implants remains challenging. A previous systematic review was performed a few years ago (Grischke et al., 2016). Thus, an updated review is necessary. Additionally, other systematic reviews have only evaluated the ability of a selected type of method for decontamination of the implant surface (Louropoulou et al., 2015; Chouirfa et al., 2018; Dutra et al., 2018; Mishra and Chowdhary, 2018). The primary objective of this systematic review was to study and report evidence on all currently investigated methods for decontaminating pathogenic microbiota on dental implant surfaces.

## Methods

### Data Sources

A systematic search was performed in Science Direct, PubMed, and Springer Link from inception up until December 2020. The search was limited to studies published in the English language due to the authors’ inability to translate other published languages in the literature. The following search terms were used: “dental” AND “implant” AND “titanium” AND “bacteria” AND “biofilm” AND (“treatment” OR “decontamination” OR “eradication” OR “cleaning” OR “remove”). Wildcards, e.g., asterisk (*), a question mark ()?, or other symbols that designate the selected database, were applied. Original papers comprising of experimental studies that used titanium materials for the studies were included in the present review. The studies included should have reported the presence of microbial biofilm both by bacteria and fungi. After date was extracted, it was entered into Microsoft Excel in a data extraction form for enabling summarization of data and final report writing. Data analysis was completed using SPSS Software Version 23 and PRISM Software Version 7 (GraphPad Inc.).

## Results and Discussion

The electronic exploration yielded six hundred and forty-three results (*n* = 643). Duplicates were removed, and on additional screening, one hundred and sixteen studies comprised this review (*n* =116) (Schwarz et al., 2005; Duarte et al., 2009; Ewald and Ihde, 2009; Größner-Schreiber et al., 2009; Sennhenn-Kirchner et al., 2009; Tamai et al., 2009; Gonçalves et al., 2010; Baffone et al., 2011; Ercan et al., 2011; Fröjd et al., 2011; Giordano et al., 2011; Mohn et al., 2011; Ntrouka et al., 2011; Bürgers et al., 2012; Cortizo et al., 2012; Lilja et al., 2012; Rehman et al., 2012; Subramanian et al., 2012; Trujillo et al., 2012; Alcheikh et al., 2013; Cochis et al., 2013; De Giglio et al., 2013; Diab Al-Radha et al., 2013; Eick et al., 2013; Holmberg et al., 2013; Idlibi et al., 2013; Roberts et al., 2013; Chen et al., 2014; Ciandrini et al., 2014; Drago et al., 2014; Godoy-Gallardo et al., 2014; Hauser-Gerspach et al., 2014; Kaliaraj et al., 2014; Kang et al., 2014; Lv et al., 2014; Massa et al., 2014; Sahrmann et al., 2014; Schmage et al., 2014; Schmidt et al., 2014; Yamada et al., 2014; Abdulkareem et al., 2015; Charalampakis et al., 2015; Cruz et al., 2015; de Avila et al., 2015; Duske et al., 2015; Janković et al., 2015; Jennings et al., 2015; Lewandowska et al., 2015; Narendrakumar et al., 2015; Park et al., 2015; Yucesoy et al., 2015; Wood et al., 2015; Zhang et al., 2015; Ayre et al., 2016; Chen et al., 2016; Ciandrini et al., 2016; Cochis et al., 2016; Cotolan et al., 2016; Cunha et al., 2016; Giannelli et al., 2016; Godoy-Gallardo et al., 2016; Gopal et al., 2016; Guan et al., 2016; John et al., 2016; Kuehl et al., 2016; Kotsakis et al., 2016; Mang et al., 2016; Preissner et al., 2016; Rodríguez-Contreras et al., 2016; Shi et al., 2016; Verardi et al., 2016; Al-Hashedi et al., 2017; Batsukh et al., 2017; Canullo et al., 2017; Ciandrini et al., 2017; Cometa et al., 2017; Dostie et al., 2017; Eick et al., 2017; Ferraris et al., 2017; Giannelli et al., 2017; Granick et al., 2017; Hirschfeld et al., 2017; Kim et al., 2017; Kulkarni Aranya et al., 2017; Li et al., 2017; Macpherson et al., 2017; Matthes et al., 2017; Prieto-Borja et al., 2017; Schmidt et al., 2017; Strever et al., 2017; Ramesh et al., 2017; Wang et al., 2017; Wiedmer et al., 2017; Ye et al., 2017; Zhang et al., 2017; Al-Hashedi et al., 2018; Akhavan et al., 2018; Atefyekta et al., 2018; Azizi et al., 2018; Ferraris et al., 2018; Fukushima et al., 2018; Hidalgo-Robatto et al., 2018; Hoyos-Nogués et al., 2018; Lee et al., 2018; Montelongo-Jauregui et al., 2018; Pantaroto et al., 2018; Pissinis et al., 2018; Santos-Coquillat et al., 2018; Schneider et al., 2018; Souza et al., 2018; Trobos et al., 2018; Vilarrasa et al., 2018; Wang et al., 2018; Zhang et al., 2018; Huang et al., 2019; Schmidt et al., 2019).

A flowchart (Supplementary Information 1) demonstrates the procedure of inclusion and exclusion of pertinent articles. We excluded review papers, non-English language studies, and those not relevant to the focus of this review. Our analysis revealed that the topic of our systematic review has been deeply researched for the previous 10 years, especially in the years 2017–2018. The interest on this topic is mainly because of titanium being an inert material with the properties of encouraging tissue healing and bacterial colonization resistance, as compared with other implant materials (Chen et al., 2017).

Numerous studies have attempted to evaluate different methods to decontaminate dental biofilms. The decontamination methods included in this systematic review were categorized into 19 groups, namely, the coating of titanium materials (47.4%), mechanical cleaning (10.3%), laser treatment (9.5%), photodynamic therapy (4.3%), air polishing (3.4%), anodizing treatment (3.4%), radiation (3.4%), sonication (0.9%), thermal treatment (0.9%), ultrasound (0.9%), chemical treatment (13.8%), electrochemical treatment (1.7%), antimicrobial drugs (0.9%), argon treatment (0.9%), and probiotics (0.9%). **Table 1** presented the decontamination methods using specialized instruments and the findings of the respective studies. These decontamination methods were then divided into two types based on whether the biofilm was already established (biofilm-treatment) or not (biofilm-prevention). Supplementary Information 2 shows the coating of the titanium materials, method of decontamination, and type of decontamination method.

**Table 1:** Decontamination methods using specialized instruments and the findings of the respective studies.

No.	Type of method	Method of decontamination	Study findings	Type of decontamination method (Biofilm-Prevention or Biofilm-Treatment)	Reference
1	Mechanical cleaning	Titanium surface treatment instruments	Metal curets are not recommended for smooth titanium surface debridement due to severe texture alteration. Rough surfaces treated with a metal curet and the air-powder abrasive system were less susceptible to bacterial adhesion, probably due to texture modification and the presence of abrasive deposits.	Biofilm-Prevention	(Duarte et al., 2009)
2	Mechanical cleaning	Stainless steel (SSC), titanium curets (TC), air-polisher using glycine-based, perio (PP), soft powders (SP), erythritol powder (EP) and an ultrasonic device using stainless steel (PS) or plastic-coated instruments (PI)	No significant differences were observed in the surface characteristics (except for SSC) or bacterial colonization based on one-time instrumentation.	Biofilm-Prevention	(Schmidt et al., 2017)
3	Mechanical cleaning	Manual plastic curet, manual carbon fiber-reinforced plastic (CFRP) curet, sonic-driven prophylaxis brush, rotating rubber cup with prophylaxis paste, sonic-driven polyether ether ketone (PEEK) plastic tip, ultrasonic-driven PEEK plastic tip, air polishing with amino acid (glycine) powder	The best cleaning effectiveness (less than 4% residual biofilm) was observed with the sonic and ultrasonic oscillating PEEK plastic tips and the Air polishing. However, the instruments were not able to completely clean the implant surfaces.	Biofilm-Treatment	(Schmage et al., 2014)
4	Mechanical cleaning	Treatment with a brush (BR), 1% oxygen/argon cold atmospheric plasma (CAP) (PL), or brushing combined with CAP (BR þ PL)	Neither a sole CAP treatment nor a sole brushing provided a completely decontaminated surface. A combined treatment of brushing and subsequent CAP treatment led to a cleaned surface, which allowed cells to grow comparable to cells on the sterile control.	Biofilm-Treatment	(Duske et al., 2015)
5	Mechanical cleaning	Mechanical brushing and cleansing with four different cleaning agents: NaCl, Decapinol, Hexident, Listerine	The combination of mechanical and chemical cleansing used in the present study was ineffective in complete removal of the biofilm from all four titanium discs.	Biofilm-Treatment	(Charalampakis et al., 2015)
6	Mechanical cleaning	Instrumentation with ultrasonic scalers	Treatment of titanium fixture surfaces with ultrasonic metal, plastic, or carbon tip significantly enhanced bacterial removal efficacy of brushing. Thorough instrumentation that can smooth the whole exposed surface may facilitate maintenance of the implants.	Biofilm-Treatment	(Park et al., 2015)
7	Mechanical cleaning	Curet debridement, normal saline irrigation, and placement in 0.12% chlorhexidine	The non-surgical treatment used in this study was not effective in removing LPS from titanium surfaces and increasing osteoblast adhesion.	Biofilm-Treatment	(Lee et al., 2018)
8	Mechanical cleaning	Titanium curets (TC), stainless steel ultrasonic tip (PS), erythritol air-polishing powder (EP), and rubber cup polishing (CON)	Bacterial grading showed low levels of bacteria with no significant differences (*p* < 0.05) within any of the groups at the two time points. Also, no significant differences were evident between the groups at either baseline or 12 months after regular preventive therapy (*p* > 0.05). The four treatment modalities in the present study are comparable and generally resulted in positive outcomes over the course of a year following attachment of superstructures to implants.	Biofilm-Treatment	(Schmidt et al., 2019)
9	Mechanical cleaning	Various side-to-side toothbrushes	Two of the tested side-to-side toothbrushes were able to reduce an *in-vitro* biofilm by noncontact brushing. The efficacy of the tested toothbrushes for noncontact biofilm removal differed significantly.	Biofilm-Treatment	(Schmidt et al., 2014)
10	Mechanical cleaning, laser	Metal curets, plastic curets, titanium brush, laser	Ti brushes were more effective than curets and Er: YAG laser in the removal of surface contaminants, whereas Er: YAG laser was more effective than curets and Ti brushes in killing the biofilm bacteria. None of the methods tested in this study was able to completely eliminate Ti surface contaminants.	Biofilm-Treatment	(Al-Hashedi et al., 2017)
11	Mechanical cleaning and laser treatment	Gracey (dentin) or titanium curets (CUR), Er:YAG, photodynamic therapy (PDT) and CUR with adjunctive PDT (CUR/PDT)	The use of Er:YAG laser yielded clear advantages compared to the other debridement modalities. All treatment methods decreased statistically significantly (*p* < 0.001) total CFUs in biofilms compared with untreated titanium surfaces.	Biofilm-Treatment	(Eick et al., 2017)
12	Mechanical cleaning and laser treatment	Ultrasonic scaler, rubber polishing cup, gallium–aluminum–arsenide laser, and chlorhexidine (CHX) rinse	Ultrasonic scaling with a metal tip significantly increased titanium surface roughness and significantly increased the adherence of *P. gingivalis* compared to those of surfaces treated with polishing, laser, and chemical debridement. Moreover, the growth of bacterial biofilm was the lowest for smoother surfaces, than polishing, CHX, and laser treated surfaces.	Biofilm-Treatment	(Batsukh et al., 2017)
13	Laser treatment	Femtosecond laser surface texturing using a Yb:KYW chirped-pulse-regenerative amplification laser system with a central wavelength of 1030 nm and a pulse duration of 500 fs	The laser treatment significantly reduced the bacterial adhesion to the surface as well as biofilm formation as compared to a reference polished surfaces. Femtosecond laser texturing is a simple and promising method for endowing titanium implants with antibacterial properties, reducing the risk of implant-associated infections without requiring immobilized antibacterial substances, nanoparticles or coatings.	Biofilm-Prevention	(Cunha et al., 2016)
14	Laser treatment	Erbium:yttrium–aluminium–garnet and diode laser light	Compared to the untreated controls Candida cells grown in mature *in-vitro* biofilms were significantly reduced by both wavelengths investigated. Comparison between the different methods of laser treatment additionally revealed a significantly greater effect of the Er:YAG over the diode laser. Scanning electron microscopy findings proved that the diode laser light was effective in direct contact mode. In contrast, in the areas without direct contact, the fungal cells were left almost unchanged. The Er:YAG laser damaged the fungal cells to a great extent wherever it was applied.	Biofilm-Prevention	(Sennhenn-Kirchner et al., 2009)
15	Laser treatment	Tissue tolerable plasma	Prior to treatment, CLSM and SEM detected adhering bacteria. Post-treatment FM recorded that the number of dead cells was higher using TTP compared to DL and C.	Biofilm-Treatment	(Preissner et al., 2016)
16	Laser treatment	Cold atmospheric plasma (CAP), diode laser (DL), air-abrasion (AA), chlorhexidine (CHX)	Treatment of oral biofilms with CAP reduced the quantity of the biofilms, destroyed their structure and reduced superficial bacteria while complete removal was not achieved. However, according to the microbiological assay, CAP produced the best results with regard to disinfection of microorganisms from the oral biofilm.	Biofilm-Treatment	(Idlibi et al., 2013)
17	Laser treatment	980-nm gallium aluminum arsenide (GaAlAs) and 1064-nm neodymium-doped yttrium aluminum garnet (Nd:YAG) lasers	At 3W, both lasers decontaminated all surfaces no matter the type of bacteria tested, whereas at 2.5 W, the rough implants were partially decontaminated. The 980-nm diode and the 1064-nm Nd:YAG lasers were effective in decontamination of *P. gingivalis* and *E. faecalis* without promoting surface alteration on the implants.	Biofilm-Treatment	(Gonçalves et al., 2010)
18	Laser treatment	Radiation from an irrigated erbium, chromium–doped yttrium, scandium, gallium, garnet (Er,Cr:YSGG laser) (Waterlase iPlus, Biolase, Irvine, CA)	Er,Cr:YSGG laser treatment of titanium disks coated with single –species biofilm of *P. gingivalis* ablates >95% of the biofilm with no physical changes noted to the implant material surface coating.	Biofilm-Treatment	(Strever et al., 2017)
19	Laser treatment	Er:YAG laser	No significant adverse effect on subsequent colonization and proliferation of MG-63 cells or increased bacterial adhesion was found in comparison to untreated control surfaces.	Biofilm-Treatment	(Hauser-Gerspach et al., 2014)
20	Laser treatment	Er:YAG laser device (KEY3, KaVo, Biberach, Germany)	An Er:YAG laser (100 mJ/pulse, 10 Hz) seems to be most suitable for the removal of supragingival early plaque biofilms grown on SLA titanium implants, and (2) all treatment procedures failed to restore the biocompatibility of previously-contaminated SLA titanium surfaces.	Biofilm-Treatment	(Schwarz et al., 2005)
21	Laser treatment	Diode laser emitting at λ 808 nm (Dental Laser System 4 × 4™, General Project Ltd., Montespertoli, Florence, Italy)	Diode laser irradiation in both continuous and pulsed modes induced a statistically significant reduction of viable bacteria and nitrite levels. These results indicate that in addition to its bactericidal effect laser irradiation can also inhibit LPS-induced macrophage activation and thus blunt the inflammatory response.	Biofilm-Treatment	(Giannelli et al., 2016)
22	Laser treatment and photodynamic therapy	Photodynamic laser treatment (PDT)	1-min treatment with λ 405 nm LED, or λ 635 nm diode laser, or 0.1% MB alone did not result in any appreciable reduction of viability of *S. aureus* biofilm, while prolongation of the treatments with λ 405 nm LED or λ 635 nm laser with MB up to 5 min resulted in a marked, statistically significant reduction of *S. aureus* biofilm.	Biofilm-Prevention	(Giannelli et al., 2017)
23	Laser treatment and photodynamic therapy	Methylene blue-mediated photodynamic therapy	MB-mediated PDT displayed the concentration-dependent, pH-dependent, and time-dependent efficacy of eradication therapy on biofilm-contaminated implant surfaces. The most effective treatment occurred with exposure to laser light in combination with the 200 μg/mL MB photosensitizer at pH 10 for 60 s of irradiation time.	Biofilm-Treatment	(Huang et al., 2019)
24	Photodynamic therapy	Photodynamic therapy and light-activated disinfection	Both PDT1 and PDT2 protocols showed a high efficacy against a 3-day-old bacterial biofilm on dental implants and were more effective compared with LAD.	Biofilm-Treatment	(Azizi et al., 2018)
25	Photodynamic therapy	Photodynamic therapy (PDT), using a diode laser at 630 nm, 150 mW/cm^2^ for light doses ranging from 25–100 J/cm^2^	A dose dependent efficacy of a PDT treatment against *F. nucleatum* grown in biofilm was significant.	Biofilm-Treatment	(Mang et al., 2016)
26	Photodynamic therapy	Photodynamic therapy using methylene blue and red color diode laser	The combination of methylene blue and a red color LED (Periowave®) was effective in reducing the viability of biofilm attached to SLA titanium surface *in-vitro*.	Biofilm-Treatment	(Kim et al., 2017)
27	Radiation	Ultraviolet-C pre-irradiation	UVC irradiation reduced the attachment or biofilm formation of wound pathogens, and promoted the hydrophilicity, and carbon elimination on various topographical titanium surfaces, rivaling or surpassing UVA irradiation in effect.	Biofilm-Prevention	(Yamada et al., 2014)
28	Radiation	UV treatment of titanium immediately prior to use (photofunctionalization)	Denaturing gradient gel-electrophoresis (DGGE) and DNA sequencing analyses revealed that while bacterial community profiles appeared different between UV-treated and untreated titanium in the initial attachment phase, this difference vanished as biofilm formation progressed.	Biofilm-Prevention	(de Avila et al., 2015)
29	Radiation	UV-treated titanium with nanonetwork structures (UV-TNS)	The surface characteristics and biological properties of TNS could be modulated via short-duration high-intensity UV treatment. UV-TNS effectively inhibited attachment and biofilm formation of *A. oris*. UV treatment could enhance both the biological compatibility and the antimicrobial activity of TNS.	Biofilm-Treatment	(Zhang et al., 2017)
30	Radiation	Photoactivated disinfection with a light-emitting diode	(a) PAD using LED is effective against periodontopathic microbial species even in the presence of serum, (b) PAD and HPAD reduce viability in single-species biofilms. (C) the increase in antimicrobial activity following the use of H-PAD may bear potential relevance as an adjunctive antimicrobial treatment in periodontal and peri-implant infections thus warranting further clinical testing.	Biofilm-Treatment	(Eick et al., 2013)
31	Sonication	Influence of exposure time of sonication	5 minutes is the optimal time of sonication in order to recover the maximum amount of most bacteria attached to Ti6Al4V discs.	Biofilm-Treatment	(Prieto-Borja et al., 2017)
32	Thermal treatment	Influence of thermally (50–800 C) stabilized titanium (TS-Ti) nano oxide	The bacterial adhesion studies revealed that the TS-Ti films formed at temperatures below 350 C were refractory to bacterial adhesion, while temperatures above 600 C showed significantly enhanced affinity towards bacterial adhesion.	Biofilm-Prevention	(Gopal et al., 2016)
33	Ultrasound treatment	Direct-contact low-frequency ultrasound	This experiment demonstrates that the DCLFU device can clear *S epidermidis* biofilms off of metallic implant material. At the moderate power levels and brief exposure time used in this study, the bacteria are converted to the planktonic state with biofilm dispersion. Using HCA as the mechanical irrigant facilitates killing of the planktonic bacteria that are released.	Biofilm-Treatment	(Granick et al., 2017)
34	Air polishing	Air polishing with erythritol/chlorhexidine or glycine and amorphous silica	Air polishing with erythritol/chlorhexidine seems to be a viable alternative to the traditional glycine treatment for biofilm removal.	Biofilm-Treatment	(Drago et al., 2014)
35	Air polishing	Air polishing with glycine powder	air-polishing treatment with glycine powder causes minimal damage to the implant surfaces and reduce bacterial recolonization in a short test period (24 h).	Biofilm-Treatment	(Cochis et al., 2013)
36	Air polishing	Optimized air polishing and a cold plasma device. Air powder contains erythritol (sugar alcohol, 14 μm), amorphous silica and 0.3 % chlorhexidine (CHX).	Air-polishing with erythritol powder has a high potential to render a microbially contaminated implant surface cell conductive. The concomitant use of air polishing and plasma treatment did not enhance osteoblast spreading.	Biofilm-Treatment	(Matthes et al., 2017)
37	Air polishing	Air-abrasive powder based on glycine and tricalcium phosphate	Glycine and tricalcium phosphate seemed to be more effective than the control groups for biofilm removal on titanium surfaces.	Biofilm-Treatment	(John et al., 2016)
38	Anodizing treatment	Effect of titanium anodization and nanotube diameter	The adherence of oral streptococci can be modified by titanium anodization and nanotube diameter.	Biofilm-Prevention	(Narendrakumar et al., 2015)
39	Anodizing treatment	Anodized nanotubular titanium and the effect of electrical stimulation	Decreased *S. aureus* biofilm formation was found after 2 days of culture on 15–30 V stimulated anodized nanotubular titanium compared with non-stimulated conventionally used titanium.	Biofilm-Prevention	(Ercan et al., 2011)
40	Anodizing treatment	Electrochemically induced anatase at two different voltages: 90 and 130 V for pure titanium; 100 and 120 V for Ti6Al4V alloy	The high voltage anodization treatments may contribute to preserving tissue integration and reduce bacteria colonization of titanium and titanium alloy for implant-based applications.	Biofilm-Prevention	(Giordano et al., 2011)
41	Anodizing treatment	Electrochemical treatment	*In-situ* generated active oxidants at the anode caused higher bacterial reduction than the mere alkaline environment emerging at the cathode.	Biofilm-Treatment	(Mohn et al., 2011)

In this systematic review, 63 studies focused on biofilm-prevention methods (*n* = 63, 54.3%) (Duarte et al., 2009; Ewald and Ihde, 2009; Größner-Schreiber et al., 2009; Sennhenn-Kirchner et al., 2009; Tamai et al., 2009; Baffone et al., 2011; Ercan et al., 2011; Fröjd et al., 2011; Giordano et al., 2011; Cortizo et al., 2012; Rehman et al., 2012; Subramanian et al., 2012; Trujillo et al., 2012; Alcheikh et al., 2013; De Giglio et al., 2013; Holmberg et al., 2013; Roberts et al., 2013; Godoy-Gallardo et al., 2014; Lv et al., 2014; Kaliaraj et al., 2014; Kang et al., 2014; Massa et al., 2014; Yamada et al., 2014; Abdulkareem et al., 2015; de Avila et al., 2015; Janković et al., 2015; Jennings et al., 2015; Lewandowska et al., 2015; Narendrakumar et al., 2015; Wood et al., 2015; Yucesoy et al., 2015; Zhang et al., 2015; Ayre et al., 2016; Cochis et al., 2016; Cotolan et al., 2016; Cunha et al., 2016; Godoy-Gallardo et al., 2016; Gopal et al., 2016; Guan et al., 2016; Kuehl et al., 2016; Rodríguez-Contreras et al., 2016; Shi et al., 2016; Ciandrini et al., 2017; Cometa et al., 2017; Ferraris et al., 2017; Giannelli et al., 2017; Hirschfeld et al., 2017; Kulkarni Aranya et al., 2017; Macpherson et al., 2017; Schmidt et al., 2017; Ye et al., 2017; Akhavan et al., 2018; Atefyekta et al., 2018; Ferraris et al., 2018; Hidalgo-Robatto et al., 2018; Hoyos-Nogués et al., 2018; Pantaroto et al., 2018; Pissinis et al., 2018; Santos-Coquillat et al., 2018; Trobos et al., 2018; Vilarrasa et al., 2018; Wang et al., 2018; Zhang et al., 2018), whereas 51 studies focused on biofilm-treatment methods (*n* = 51, 44.0%) (Schwarz et al., 2005; Gonçalves et al., 2010; Mohn et al., 2011; Ntrouka et al., 2011; Bürgers et al., 2012; Cochis et al., 2013; Diab Al-Radha et al., 2013; Eick et al., 2013; Idlibi et al., 2013; Chen et al., 2014; Drago et al., 2014; Hauser-Gerspach et al., 2014; Sahrmann et al., 2014; Schmage et al., 2014; Schmidt et al., 2014; Charalampakis et al., 2015; Cruz et al., 2015; Duske et al., 2015; Park et al., 2015; Chen et al., 2016; Ciandrini et al., 2016; Mang et al., 2016; Giannelli et al., 2016; John et al., 2016; Kotsakis et al., 2016; Preissner et al., 2016; Verardi et al., 2016; Al-Hashedi et al., 2017; Batsukh et al., 2017; Canullo et al., 2017; Dostie et al., 2017; Eick et al., 2017; Granick et al., 2017; Kim et al., 2017; Li et al., 2017; Matthes et al., 2017; Prieto-Borja et al., 2017; Ramesh et al., 2017; Strever et al., 2017; Wang et al., 2017; Wiedmer et al., 2017; Zhang et al., 2017; Al-Hashedi et al., 2018; Azizi et al., 2018; Fukushima et al., 2018; Lee et al., 2018; Montelongo-Jauregui et al., 2018; Schneider et al., 2018; Souza et al., 2018; Huang et al., 2019; Schmidt et al., 2019), while two studies offered their novel method to work for both biofilm-prevention and biofilm-treatment measures (*n* = 2, 1.7%) (Lilja et al., 2012; Ciandrini et al., 2014).

### *In Vivo* Studies

Twenty-two studies applied oral normal flora in human participants who were either healthy or diagnosed with peri-implantitis or periodontitis (*n* = 22, 19.0%) (Schwarz et al., 2005; Größner-Schreiber et al., 2009; Rehman et al., 2012; Diab Al-Radha et al., 2013; Cochis et al., 2013; Idlibi et al., 2013; Abdulkareem et al., 2015; Charalampakis et al., 2015; de Avila et al., 2015; Duske et al., 2015; Park et al., 2015; John et al., 2016; Kotsakis et al., 2016; Verardi et al., 2016; Al-Hashedi et al., 2017; Dostie et al., 2017; Li et al., 2017; Matthes et al., 2017; Al-Hashedi et al., 2018; Wang et al., 2018; Schmidt et al., 2019). One hundred and five studies performed *in-vitro* experiments (*n* = 105, 90.5%) (Duarte et al., 2009; Ewald and Ihde, 2009; Sennhenn-Kirchner et al., 2009; Tamai et al., 2009; Gonçalves et al., 2010; Baffone et al., 2011; Ercan et al., 2011; Fröjd et al., 2011; Mohn et al., 2011; Ntrouka et al., 2011; Bürgers et al., 2012; Cortizo et al., 2012; Lilja et al., 2012; Subramanian et al., 2012; Trujillo et al., 2012; Alcheikh et al., 2013; De Giglio et al., 2013; Diab Al-Radha et al., 2013; Eick et al., 2013; Holmberg et al., 2013; Roberts et al., 2013; Chen et al., 2014; Ciandrini et al., 2014; Drago et al., 2014; Godoy-Gallardo et al., 2014; Hauser-Gerspach et al., 2014; Kaliaraj et al., 2014; Kang et al., 2014; Lv et al., 2014; Massa et al., 2014; Sahrmann et al., 2014; Schmidt et al., 2014; Schmage et al., 2014; Yamada et al., 2014; Abdulkareem et al., 2015; Cruz et al., 2015; de Avila et al., 2015; Duske et al., 2015; Janković et al., 2015; Jennings et al., 2015; Lewandowska et al., 2015; Narendrakumar et al., 2015; Park et al., 2015; Wood et al., 2015; Yucesoy et al., 2015; Zhang et al., 2015; Ayre et al., 2016; Chen et al., 2016; Ciandrini et al., 2016; Cochis et al., 2016; Cotolan et al., 2016; Cunha et al., 2016; Giannelli et al., 2016; Godoy-Gallardo et al., 2016; Gopal et al., 2016; Guan et al., 2016; Kotsakis et al., 2016; Mang et al., 2016; Preissner et al., 2016; Rodríguez-Contreras et al., 2016; Shi et al., 2016; Verardi et al., 2016; Batsukh et al., 2017; Canullo et al., 2017; Ciandrini et al., 2017; Cometa et al., 2017; Dostie et al., 2017; Eick et al., 2017; Ferraris et al., 2017; Giannelli et al., 2017; Granick et al., 2017; Hirschfeld et al., 2017; Kim et al., 2017; Kulkarni Aranya et al., 2017; Li et al., 2017; Macpherson et al., 2017; Matthes et al., 2017; Prieto-Borja et al., 2017; Ramesh et al., 2017; Schmidt et al., 2017; Strever et al., 2017; Wang et al., 2017; Wiedmer et al., 2017; Ye et al., 2017; Zhang et al., 2017; Akhavan et al., 2018; Atefyekta et al., 2018; Azizi et al., 2018; Ferraris et al., 2018; Fukushima et al., 2018; Hidalgo-Robatto et al., 2018; Hoyos-Nogués et al., 2018; Lee et al., 2018; Montelongo-Jauregui et al., 2018; Pantaroto et al., 2018; Pissinis et al., 2018; Santos-Coquillat et al., 2018; Schneider et al., 2018; Souza et al., 2018; Trobos et al., 2018; Vilarrasa et al., 2018; Wang et al., 2018; Zhang et al., 2018; Huang et al., 2019), 11 studies performed *in-vivo* experiments on humans (*n* = 11, 9.5%) (Schwarz et al., 2005; Größner-Schreiber et al., 2009; Sennhenn-Kirchner et al., 2009; Baffone et al., 2011; Fröjd et al., 2011; Rehman et al., 2012; Cochis et al., 2013; Idlibi et al., 2013; Charalampakis et al., 2015; Cruz et al., 2015; John et al., 2016; Al-Hashedi et al., 2017; Al-Hashedi et al., 2018; Schmidt et al., 2019), while two studies performed both *in-vitro* and *in-vivo* experiments (*n* = 2, 1.7%) (Giordano et al., 2011; Kuehl et al., 2016). Amongst the *in-vivo* experiments which recruited human volunteers, 11 studies used titanium discs (*n* = 11, 9.5%) (Schwarz et al., 2005; Größner-Schreiber et al., 2009; Giordano et al., 2011; Rehman et al., 2012; Cochis et al., 2013; Idlibi et al., 2013; Charalampakis et al., 2015; Kuehl et al., 2016; Al-Hashedi et al., 2017; Al-Hashedi et al., 2018), while the other two studies used titanium implants (*n* = 2, 1.7%) (John et al., 2016; Schmidt et al., 2019).

### *In Vitro* Studies

Out of 116 studies, some studies did not use discs (*n* = 13, 11.2%) (Gonçalves et al., 2010; Mohn et al., 2011; Subramanian et al., 2012; Kaliaraj et al., 2014; Lewandowska et al., 2015; Narendrakumar et al., 2015; Park et al., 2015; John et al., 2016; Preissner et al., 2016; Cometa et al., 2017; Schmidt et al., 2017; Azizi et al., 2018; Schmidt et al., 2019). One study performed on discs and other form of titanium (*n* = 1, 0.9%) (Kulkarni et al., 2017), four studies did not state whether they used discs (*n* = 4, 3.4%) (Cunha et al., 2016; Macpherson et al., 2017; Ramesh et al., 2017; Akhavan et al., 2018), while the others used discs (*n* = 98, 84.5%) (Schwarz et al., 2005; Duarte et al., 2009; Ewald and Ihde, 2009; Größner-Schreiber et al., 2009; Sennhenn-Kirchner et al., 2009; Tamai et al., 2009; Baffone et al., 2011; Ercan et al., 2011; Fröjd et al., 2011; Ntrouka et al., 2011; Bürgers et al., 2012; Cortizo et al., 2012; Lilja et al., 2012; Rehman et al., 2012; Trujillo et al., 2012; Alcheikh et al., 2013; Cochis et al., 2013; De Giglio et al., 2013; Diab Al-Radha et al., 2013; Eick et al., 2013; Holmberg et al., 2013; Idlibi et al., 2013; Roberts et al., 2013; Chen et al., 2014; Ciandrini et al., 2014; Drago et al., 2014; Godoy-Gallardo et al., 2014; Hauser-Gerspach et al., 2014; Kang et al., 2014; Lv et al., 2014; Massa et al., 2014; Sahrmann et al., 2014; Schmidt et al., 2014; Schmage et al., 2014; Yamada et al., 2014; Abdulkareem et al., 2015; Charalampakis et al., 2015; Cruz et al., 2015; de Avila et al., 2015; Duske et al., 2015; Janković et al., 2015; Jennings et al., 2015; Wood et al., 2015; Yucesoy et al., 2015; Zhang et al., 2015; Ayre et al., 2016; Chen et al., 2016; Cochis et al., 2016; Ciandrini et al., 2016; Cotolan et al., 2016; Giannelli et al., 2016; Godoy-Gallardo et al., 2016; Gopal et al., 2016; Guan et al., 2016; Kotsakis et al., 2016; Kuehl et al., 2016; Mang et al., 2016; Rodríguez-Contreras et al., 2016; Shi et al., 2016; Verardi et al., 2016; Al-Hashedi et al., 2017; Batsukh et al., 2017; Canullo et al., 2017; Ciandrini et al., 2017; Dostie et al., 2017; Eick et al., 2017; Ferraris et al., 2017; Granick et al., 2017; Giannelli et al., 2017; Hirschfeld et al., 2017; Kim et al., 2017; Kulkarni Aranya et al., 2017; Li et al., 2017; Matthes et al., 2017; Prieto-Borja et al., 2017; Strever et al., 2017; Wang et al., 2017; Wiedmer et al., 2017; Ye et al., 2017; Zhang et al., 2017; Al-Hashedi et al., 2018; Atefyekta et al., 2018; Ferraris et al., 2018; Fukushima et al., 2018; Hidalgo-Robatto et al., 2018; Hoyos-Nogués et al., 2018; Huang et al., 2019; Lee et al., 2018; Montelongo-Jauregui et al., 2018; Pantaroto et al., 2018; Pissinis et al., 2018; Santos-Coquillat et al., 2018; Schneider et al., 2018; Souza et al., 2018; Trobos et al., 2018; Vilarrasa et al., 2018; Wang et al., 2018; Zhang et al., 2018).

### Studies With Development of Dental Biofilm

Forty-nine different types of microorganisms have been used for the development of dental biofilm in the included studies for this systematic review (*n* = 49, **Table 2**). The biofilm matrix was either in single species or mixed species. Seventy-eight studies performed on single-species biofilm (*n* = 78, 67.2%) (Duarte et al., 2009; Ewald and Ihde, 2009; Sennhenn-Kirchner et al., 2009; Tamai et al., 2009; Gonçalves et al., 2010; Ercan et al., 2011; Giordano et al., 2011; Trujillo et al., 2012; Bürgers et al., 2012; Lilja et al., 2012; Roberts et al., 2013; Holmberg et al., 2013; De Giglio et al., 2013; Alcheikh et al., 2013; Chen et al., 2014; Drago et al., 2014; Godoy-Gallardo et al., 2014; Hauser-Gerspach et al., 2014; Kaliaraj et al., 2014; Kang et al., 2014; Lv et al., 2014; Massa et al., 2014; Schmage et al., 2014; Yamada et al., 2014; Zhang et al., 2015; Jennings et al., 2015; Narendrakumar et al., 2015; Yucesoy et al., 2015; Wood et al., 2015; Janković et al., 2015; Lewandowska et al., 2015; Ayre et al., 2016; Chen et al., 2016; Ciandrini et al., 2016; Cotolan et al., 2016; Cunha et al., 2016; Giannelli et al., 2016; Godoy-Gallardo et al., 2016; Gopal et al., 2016; Guan et al., 2016; Kuehl et al., 2016; Mang et al., 2016; Preissner et al., 2016; Rodríguez-Contreras et al., 2016; Shi et al., 2016; Batsukh et al., 2017; Canullo et al., 2017; Ciandrini et al., 2017; Cometa et al., 2017; Eick et al., 2017; Ferraris et al., 2017; Giannelli et al., 2017; Granick et al., 2017; Hirschfeld et al., 2017; Kim et al., 2017; Kulkarni Aranya et al., 2017; Macpherson et al., 2017; Prieto-Borja et al., 2017; Strever et al., 2017; Zhang et al., 2017; Wang et al., 2017; Wiedmer et al., 2017; Ye et al., 2017; Akhavan et al., 2018; Atefyekta et al., 2018; Azizi et al., 2018; Ferraris et al., 2018; Fukushima et al., 2018; Hidalgo-Robatto et al., 2018; Hoyos-Nogués et al., 2018; Lee et al., 2018; Pissinis et al., 2018; Santos-Coquillat et al., 2018; Souza et al., 2018; Trobos et al., 2018; Zhang et al., 2018; Huang et al., 2019). Ten studies performed on mixed-species biofilm (*n* = 10, 8.6%) (Fröjd et al., 2011; Cortizo et al., 2012; Subramanian et al., 2012; Sahrmann et al., 2014; Schmidt et al., 2014; Cruz et al., 2015; Cochis et al., 2016; Ramesh et al., 2017; Schneider et al., 2018; Vilarrasa et al., 2018), while eight studies compared single-species and mixed species biofilm (*n* = 8, 6.9%) (Baffone et al., 2011; Ntrouka et al., 2011; Eick et al., 2013; Ciandrini et al., 2014; Annunziata et al., 2017; Schmidt et al., 2017; Montelongo-Jauregui et al., 2018; Pantaroto et al., 2018). Decontamination methods using antimicrobial drugs, chemical treatment, electrochemical treatment, probiotic, and the findings of the respective studies are presented in Supplementary Information 3.

**Table 2:** List of microbiota used for the development of biofilm on the titanium materials.

No.	Microbiota	Reference number	Incubation time	Reference
1	*Acinetobacter baumannii*	DSM 30007	72 h	(Cochis et al., 2016)
2	*Actinomyces naeslundii*	Clinical patient	2, 14 h	(Fröjd et al., 2011)
		ATCC 12104	48 h	(Eick et al., 2013)
			48, 72 h	(Eick et al., 2017)
			24 h	(Schmidt et al., 2017)
		OMZ 745	16.5 h	(Pantaroto et al., 2018)
3	*Actinomyces oris*	OMZ 745	40.5 h	(Sahrmann et al., 2014)
		MG-1	6 h	(Zhang et al., 2017)
4	*Actinomyces viscosus*	ATCC 15987	2 d	(Vilarrasa et al., 2018)
		ATCC 19246	14 d	(Zhang et al., 2015)
5	*Aggregatibacter actinomycetemcomitans*	ATCC 33384	72 h	(Azizi et al., 2018)
			72 h	(Kim et al., 2017)
		IDH 781	24 h	(Huang et al., 2019)
		Serotype b	1, 2, 4, 6 d	(Massa et al., 2014)
		VT 1169	5 d	(Ramesh et al., 2017)
		Y4	48 h	(Eick et al., 2013)
			72 h	(Lee et al., 2018)
			48, 72 h	(Eick et al., 2017)
6	*Bacillus atrophaeus*	ATCC 9372	1, 3, 7 d	(Ewald and Ihde, 2009)
7	*Bacillus megaterium*	n/s	10 d	(Subramanian et al., 2012)
8	*Bacillus pseudomycoides*	n/s	10 d	(Subramanian et al., 2012)
9	*Bacteroides fragilis*	n/s	48 h	(Drago et al., 2014)
10	*Campylobacter rectus*	ATCC 33238	48 h	(Eick et al., 2013)
			48, 72 h	(Eick et al., 2017)
		OMZ 698	40.5 h	(Sahrmann et al., 2014)
11	*Candida albicans*	Clinical patient	5 d	(Sennhenn-Kirchner et al., 2009)
		Clinical patient	8 d	(Cruz et al., 2015)
		n/s	48 h	(Drago et al., 2014)
		SC5314	24 h	(Montelongo-Jauregui et al., 2018)
		ATCC 10231	120 mins	(Bürgers et al., 2012)
		ATCC 90028	4, 18, 24 h	(Akhavan et al., 2018)
12	*Candida famata*	30	24, 48, 72 h	(Janković et al., 2015)
13	*Eikenella corrodens*	ATCC 23834	48 h	(Eick et al., 2013)
			48, 72 h	(Eick et al., 2017)
14	*Enterobacter cloacae*	ATCC 13047	24 h	(Kuehl et al., 2016)
		ATCC 23355	24 h	(Kuehl et al., 2016)
15	*Enterococcus faecalis*	ATCC 29212	24 h	(Chen et al., 2016)
			24 h	(Prieto-Borja et al., 2017)
			18 h	(Gonçalves et al., 2010)
16	*Escherichia coli*	ATCC 2592	7, 24, 30 h	(De Giglio et al., 2013)
			24 h	(Prieto-Borja et al., 2017)
		ATCC DH5 ALPHA	24 h	(Chen et al., 2016)
		C43	60 h	(Mohn et al., 2011)
		HB 101	48 h	(Schneider et al., 2018)
		K12 JM 101	48 h	(Schneider et al., 2018)
		n/s	2 h	(Rodríguez-Contreras et al., 2016)
		n/s	24 h	(Macpherson et al., 2017)
		NCIM 2931	6 h	(Gopal et al., 2016)
17	*Eubacterium nodatum*	ATCC 33099	48 h	(Eick et al., 2013)
18	*Filifactor alocis*	ATCC 35896	48, 72 h	(Eick et al., 2017)
19	*Fusarium solani*	ATCC 36031	1, 3, 7 d	(Ewald and Ihde, 2009)
20	*Fusobacterium nucleatum*	ATCC 10953	72 h	(Schmidt et al., 2014)
		ATCC 25586	2 d	(Vilarrasa et al., 2018)
			24 h	(Kang et al., 2014)
			24 h	(Mang et al., 2016)
			48 h	(Eick et al., 2013)
			16.5, 40.5, 64.5 h	(Ciandrini et al., 2014)
			48, 72 h	(Eick et al., 2017)
			24 h	(Schmidt et al., 2017)
		OMZ 596	16.5 h	(Pantaroto et al., 2018)
		OMZ 598	40.5 h	(Sahrmann et al., 2014)
21	*Klebsiella pneumoniae*	ATCC 4352	24 h	(Tamai et al., 2009)
22	*Lactobacillus acidophilus*	ATCC 4356	24 h	(Ciandrini et al., 2017)
		DDS-1	24 h	(Ciandrini et al., 2017)
23	*Lactobacillus casei*	ATCC 15008	24 h	(Ciandrini et al., 2017)
24	*Lactobacillus paracasei*	B21060	24 h	(Ciandrini et al., 2017)
25	*Lactobacillus reuteri*	DSM 17938	24 h	(Ciandrini et al., 2017)
26	*Lactobacillus rhamnosus*	ATCC 53103	24 h	(Ciandrini et al., 2017)
		ATCC 7469	24 h	(Ciandrini et al., 2017)
27	*Lactobacillus salivarius*	CCUG 17826	2 h	(Godoy-Gallardo et al., 2014)
			2, 24 h	(Godoy-Gallardo et al., 2016)
		ATCC 11741	24 h	(Ciandrini et al., 2017)
28	*Lysinibacillus sphaericus*	n/s	24 h	(Kaliaraj et al., 2014)
29	*Parvimonas micra*	ATCC 33270	48 h	(Eick et al., 2013)
			48, 72 h	(Eick et al., 2017)
30	*Porphyromonas gingivalis*	381	48 h	(Strever et al., 2017)
		A7436	24 h	(Chen et al., 2016)
			24 h	(Huang et al., 2019)
		ATCC 33277	2 h	(Hauser-Gerspach et al., 2014)
			14 d	(Zhang et al., 2015)
			72 h	(Lee et al., 2018)
			2 h	(Roberts et al., 2013)
			72 h	(Azizi et al., 2018)
			2 d	(Vilarrasa et al., 2018)
			48 h	(Eick et al., 2013)
			8 d	(Holmberg et al., 2013)
			6, 12, 24 h	(Batsukh et al., 2017)
			3, 7 d	(Kulkarni Aranya et al., 2017)
			16.5, 40.5, 64.5 h	(Ciandrini et al., 2014)
			40.5 h	(Sahrmann et al., 2014)
			24 h	(Kang et al., 2014)
			24 h	(Schmidt et al., 2017)
			3 d	(Gonçalves et al., 2010)
		ATCC BAA-308	24 h	(Jennings et al., 2015)
		CCUG 25211	24 h	(Giordano et al., 2011)
		DSM 20709	72 h	(Schmidt et al., 2014)
31	*Prevotella denticola*	ATCC 35308	24 h	(Jennings et al., 2015)
32	*Prevotella intermedia*	Clinical patient	72 h	(Azizi et al., 2018)
		ATCC 25611	48 h	(Eick et al., 2013)
			48, 72 h	(Eick et al., 2017)
			40.5 h	(Sahrmann et al., 2014)
33	*Pseudomonas aeruginosa*	ATCC 27853	24 h	(Prieto-Borja et al., 2017)
			24 h	(Cotolan et al., 2016)
			7, 24, 30 h	(De Giglio et al., 2013)
			24, 48, 72 h	(Janković et al., 2015)
		ATCC 9027	1, 3, 7 d	(Ewald and Ihde, 2009)
			16.5, 40.5, 64.5 h	(Baffone et al., 2011)
		CCUG 10778	24, 48 h	(Atefyekta et al., 2018)
		DSM939	24, 48 h	(Cometa et al., 2017)
		NBRC 3080	24 h	(Tamai et al., 2009)
		PA 01	8, 24, 32, 48 h	(Trujillo et al., 2012)
34	*Staphylococcus aureus*	38	4, 18, 24 h	(Akhavan et al., 2018)
		15981	24 h	(Hidalgo-Robatto et al., 2018)
			24 h	(Prieto-Borja et al., 2017)
		H9(Clinical patient)	24 h	(Lewandowska et al., 2015)
		ATCC 12598 (Cowan I strain)	1 h	(Alcheikh et al., 2013)
		ATCC 12600	24 h	(Kuehl et al., 2016)
		ATCC 13420(Newman)	24 h	(Kuehl et al., 2016)
		ATCC 25923	24 h	(Jennings et al., 2015)
			1 hr, 4 hr, 8 hr, 1 d, 2 d	(Ercan et al., 2011)
			24 h	(Wang et al., 2017)
			2 h	(Pissinis et al., 2018)
			48 h	(Giannelli et al., 2017)
			48 h	(Giannelli et al., 2016)
			48 h	(Cunha et al., 2016)
		ATCC 29213	24 h	(Ferraris et al., 2018)
			7, 24, 30 h	(De Giglio et al., 2013)
			24 h	(Lewandowska et al., 2015)
		ATCC 35556(SA113)	24 h	(Kuehl et al., 2016)
		ATCC 43387	16.5, 40.5, 64.5 h	(Baffone et al., 2011)
		ATCC 6533	24, 48, 72 h	(Janković et al., 2015)
		ATCC 6538	1, 3, 7 d	(Ewald and Ihde, 2009)
			1 min, 1 h, 4 h, 8 h	(Yamada et al., 2014)
		CCUG 56489	24, 48 h	(Atefyekta et al., 2018)
		DSM 799	24, 48 h	(Cometa et al., 2017)
		n/s	1, 3, 5, 7, 14 d	(Lv et al., 2014)
		n/s	4, 24, 5 d	(Zhang et al., 2018)
		n/s	24 h	(Santos-Coquillat et al., 2018)
		n/s	12 h	(Shi et al., 2016)
		n/s	48 h	(Drago et al., 2014)
		n/s	24, 48, 72, 96 h	(Ayre et al., 2016)
		n/s	6, 12, 18, 24 h	(Ye et al., 2017)
		Clinical patient	90 mins, 24 h	(Ferraris et al., 2017)
		NBRC 12732	24 h	(Tamai et al., 2009)
		NCIM 5021	6 h	(Gopal et al., 2016)
		NTCC 8325-4	24 h	(Giordano et al., 2011)
35	*Staphylococcus aureus MRSA*	ATCC 43300	24 h	(Kuehl et al., 2016)
		USA 300 JE2	24 h	(Kuehl et al., 2016)
36	*Staphylococcus epidermidis*	Xen 43	8 h	(Wiedmer et al., 2017)
		RP62A	24 h	(Granick et al., 2017)
			8, 24, 32, 48 h	(Trujillo et al., 2012)
		CCUG 18000A	14 h	(Lilja et al., 2012)
		ATCC 35984	24 h	(Giordano et al., 2011)
			24, 48 h	(Atefyekta et al., 2018)
			24 h	(Hirschfeld et al., 2017)
			24 h	(Prieto-Borja et al., 2017)
		ATCC 29886	2 h	(Yucesoy et al., 2015)
		AF270147	120 mins	(Bürgers et al., 2012)
		35984	24 h	(Hidalgo-Robatto et al., 2018)
		1457	24 h	(Kuehl et al., 2016)
37	*Staphylococcus epidermidis MRSE*	ATCC 35984	24 h	(Kuehl et al., 2016)
38	*Streptococcus anginosus*	ATCC 9895(OMZ 871)	40.5 h	(Sahrmann et al., 2014)
39	*Streptococcus gordonii*	ATCC 10558	48 h	(Eick et al., 2013)
			48, 72 h	(Eick et al., 2017)
			2, 24 h	(Schmidt et al., 2017)
			24 h	(Schmidt et al., 2017)
		DL1	0, 8, 24, 48 h	(Wood et al., 2015)
		DL1.1	24 h	(Montelongo-Jauregui et al., 2018)
		ML-5	72 h	(Chen et al., 2014)
		n/s	6, 24 h	(Guan et al., 2016)
40	*Streptococcus mitis*	n/s	2, 7 d	(Cortizo et al., 2012)
		n/s	2 h	(Canullo et al., 2017)
		BA7	8 h	(Dorkhan et al., 2014)
		DSM 12643	24 h	(Preissner et al., 2016)
41	*Streptococcus mutans*	3209	1 h	(Narendrakumar et al., 2015)
		ATCC 25175	24 h	(Ciandrini et al., 2017)
			8 d	(Cruz et al., 2015)
			16.5, 40.5, 64.5 h	(Ciandrini et al., 2014)
			16.5, 40.5, 64.5 h	(Baffone et al., 2011)
			24 h	(Ciandrini et al., 2016)
			2 h	(Roberts et al., 2013)
			2 h	(Yucesoy et al., 2015)
		ATCC 700610	24 h	(Huang et al., 2019)
		C180-2(Clinical patient)	8, 24, 32 h	(Ntrouka et al., 2011)
		CCUG 35176	24 h	(Giordano et al., 2011)
		n/s	n/s	(Schmage et al., 2014)
		n/s	6, 12, 18, 24 h	(Ye et al., 2017)
		NCTC 10449	30, 90 mins	(Fukushima et al., 2018)
		UA 159	14 d	(Zhang et al., 2015)
			5 d	(Ramesh et al., 2017)
42	*Streptococcus oralis*	ATCC 35037(CCUG 13229 T)	2, 24, 48 h	(Trobos et al., 2018)
		ATCC 6249	2 d	(Vilarrasa et al., 2018)
		ATCC 9811	24 h	(Ciandrini et al., 2017)
			24 h	(Ciandrini et al., 2016)
		SK248(OMZ 607)	40.5 h	(Sahrmann et al., 2014)
43	*Streptococcus pyogenes*	GTC 262	1 min, 1 h, 4 h, 8 h	(Yamada et al., 2014)
44	*Streptococcus salivarius*	n/s	2, 7 d	(Cortizo et al., 2012)
		ATCC 13419	5 d	(Ramesh et al., 2017)
45	*Streptococcus sanguinis*	ATCC 10556	24 h	(Chen et al., 2016)
			16, 48 h	(Duarte et al., 2009)
			72 h	(Kim et al., 2017)
			2, 14 h	(Fröjd et al., 2011)
			5 d	(Ramesh et al., 2017)
		CECT 480	4 h	(Hoyos-Nogués et al., 2018)
			2 h	(Godoy-Gallardo et al., 2014)
			2, 24 h	(Godoy-Gallardo et al., 2016)
		DSM 20068	2 h	(Bürgers et al., 2012; Hauser-Gerspach et al., 2014)
			72 h	(Schmidt et al., 2014)
		GW2	1 h	(Narendrakumar et al., 2015)
		IAL 1832	16.5 h	(Pantaroto et al., 2018)
			1.5 h	(Souza et al., 2018)
		n/s	6, 24 h	(Guan et al., 2016)
46	*Tannerella forsythia*	ATCC 43037	48 h	(Eick et al., 2013)
			48, 72 h	(Eick et al., 2017)
			24 h	(Schmidt et al., 2017)
47	*Treponema denticola*	ATCC 35405	48 h	(Eick et al., 2013)
			48, 72 h	(Eick et al., 2017)
48	*Veillonella dispar*	ATCC 17748T	40.5 h	(Sahrmann et al., 2014)
49	*Veillonella parvula*	ATCC 10790	2 d	(Vilarrasa et al., 2018)

n/s, not stated; DSM/DSMZ, Deutsche Sammlung von Mikroorganismen und Zellkulturen GmbH; ATCC, American Tissue Culture Collections; OMZ, Institut für Orale Mikrobiologie und Allgemeine Immunologie; NCIM, National Collection of Industrial Microorganisms, National Chemical Laboratory; CCUG, Culture Collection, University of Göteborg, Department of Clinical Bacteriology, Institute of Clinical Bacteriology, Immunology, and Virology, Sweden; NBRC, Biological Resource Center, National Institute of Technology and Evaluation, Japan; NTCC, Culture Collection, Microbiology and Cell Biology Laboratory, Indian Institute of Science; CECT, Colección Espagñola de Cultivos Tipo, Universitat de Valencia, Edeficio de Investigación, Spain; h, hours; Mins, minutes; D, days.

In experiments that used bacteria or fungi either as single species or mixed species (without the exposure to normal flora), 143 experiments applied biofilm aged less or equal to 24 h (*n* = 143, 64.7%), 72 experiments applied biofilm aged more than 24 h or equal to 48 h (*n* = 72, 32.6%), 38 experiments applied biofilm aged more than 48 h or equal to 72 h (*n* = 38, 17.2%), while 23 experiments applied biofilm aged more than 72 h (*n* = 23, 10.4%) (**Table 2**). **Table 2** shows the list of oral microbiota used for the development of biofilm on the titanium materials.

The included studies in this systematic review applied several methods after decontamination of the titanium material either to quantify the microbial biofilm or to observe the morphological changes. Fifty-three studies used the number of colony forming unit (CFU) (*n* = 53, 45.7%) (Duarte et al., 2009; Ewald and Ihde, 2009; Tamai et al., 2009; Gonçalves et al., 2010; Giordano et al., 2011; Baffone et al., 2011; Mohn et al., 2011; Ntrouka et al., 2011; Cochis et al., 2013; Diab Al-Radha et al., 2013; Eick et al., 2013; Holmberg et al., 2013; Roberts et al., 2013; Chen et al., 2014; Ciandrini et al., 2014; Drago et al., 2014; Godoy-Gallardo et al., 2014; Hauser-Gerspach et al., 2014; Massa et al., 2014; Sahrmann et al., 2014; Abdulkareem et al., 2015; Charalampakis et al., 2015; Janković et al., 2015; Narendrakumar et al., 2015; Wood et al., 2015; Chen et al., 2016; Cochis et al., 2016; Giannelli et al., 2016; Guan et al., 2016; Kuehl et al., 2016; Mang et al., 2016; Preissner et al., 2016; Rodríguez-Contreras et al., 2016; Verardi et al., 2016; Ciandrini et al., 2017; Cometa et al., 2017; Dostie et al., 2017; Eick et al., 2017; Ferraris et al., 2017; Giannelli et al., 2017; Granick et al., 2017; Hirschfeld et al., 2017; Kim et al., 2017; Prieto-Borja et al., 2017; Wiedmer et al., 2017; Akhavan et al., 2018; Azizi et al., 2018; Ferraris et al., 2018; Pantaroto et al., 2018; Pissinis et al., 2018; Souza et al., 2018; Trobos et al., 2018; Vilarrasa et al., 2018). Fifty-eight studies used scanning electron microscopy (SEM) (*n* = 58, 50%) (Duarte et al., 2009; Sennhenn-Kirchner et al., 2009; Gonçalves et al., 2010; Ercan et al., 2011; Giordano et al., 2011; Trujillo et al., 2012; Cochis et al., 2013; Eick et al., 2013; Holmberg et al., 2013; Yamada et al., 2014; Duske et al., 2015; Godoy-Gallardo et al., 2014; Kaliaraj et al., 2014; Abdulkareem et al., 2015; Charalampakis et al., 2015; Cruz et al., 2015; de Avila et al., 2015; Lewandowska et al., 2015; Park et al., 2015; Zhang et al., 2015 ; Preissner et al., 2016; Chen et al., 2016; Cochis et al., 2016; Cotolan et al., 2016; Cunha et al., 2016; Giannelli et al., 2016; John et al., 2016; Kotsakis et al., 2016; Mang et al., 2016; Shi et al., 2016; Al-Hashedi et al., 2017; Batsukh et al., 2017; Canullo et al., 2017; Dostie et al., 2017; Giannelli et al., 2017; Kim et al., 2017; Kulkarni Aranya et al., 2017; Li et al., 2017; Matthes et al., 2017; Ramesh et al., 2017; Schmidt et al., 2017; Strever et al., 2017; Wang et al., 2017; Wiedmer et al., 2017; Zhang et al., 2017; Al-Hashedi et al., 2018; Atefyekta et al., 2018; Azizi et al., 2018; Hidalgo-Robatto et al., 2018; Lee et al., 2018; Montelongo-Jauregui et al., 2018; Pantaroto et al., 2018; Souza et al., 2018; Trobos et al., 2018; Vilarrasa et al., 2018; Wang et al., 2018; Zhang et al., 2018; Huang et al., 2019). Thirty-two studies used confocal laser scanning microscopy (CLSM) (*n* = 32, 27.6%) (Fröjd et al., 2011; Eick et al., 2013; Drago et al., 2014; Lv et al., 2014; Sahrmann et al., 2014; Schmidt et al., 2014; Abdulkareem et al., 2015; de Avila et al., 2015; Zhang et al., 2015; Guan et al., 2016; Kotsakis et al., 2016; Preissner et al., 2016; Dostie et al., 2017; Giannelli et al., 2017; Kim et al., 2017; Kulkarni Aranya et al., 2017; Li et al., 2017; Matthes et al., 2017; Strever et al., 2017; Wiedmer et al., 2017; Ye et al., 2017; Zhang et al., 2017; Al-Hashedi et al., 2018; Akhavan et al., 2018; Hoyos-Nogués et al., 2018; Montelongo-Jauregui et al., 2018; Souza et al., 2018; Trobos et al., 2018; Wang et al., 2018; Vilarrasa et al., 2018; Zhang et al., 2018). A few of the studies that reported the use of SEM disclosed that their SEM protocol was operated between 1 and 30 kV accelerating voltage (46.7%) (Sennhenn-Kirchner et al., 2009; Ercan et al., 2011; Trujillo et al., 2012; Cochis et al., 2013; ; Eick et al., 2013; Godoy-Gallardo et al., 2014; Cruz et al., 2015; Duske et al., 2015; Lewandowska et al., 2015; Park et al., 2015; Cotolan et al., 2016; Preissner et al., 2016; Al-Hashedi et al., 2017; Batsukh et al., 2017; Dostie et al., 2017; Kulkarni Aranya et al., 2017; Matthes et al., 2017; Ramesh et al., 2017; Al-Hashedi et al., 2018; Atefyekta et al., 2018; Lee et al., 2018; Pantaroto et al., 2018; Trobos et al., 2018; Wang et al., 2018).

In this review, the most commonly employed oral microbiota for the study of dental biofilms were *Staphylococcus aureus* (15.9%) (Ewald and Ihde, 2009; Tamai et al., 2009; Baffone et al., 2011; Ercan et al., 2011; Giordano et al., 2011; Alcheikh et al., 2013; De Giglio et al., 2013; Drago et al., 2014; Lv et al., 2014; Yamada et al., 2014; Janković et al., 2015; Jennings et al., 2015; Lewandowska et al., 2015; Ayre et al., 2016; Cunha et al., 2016; Giannelli et al., 2016; Gopal et al., 2016; Kuehl et al., 2016; Shi et al., 2016; Cometa et al., 2017; Ferraris et al., 2017; Giannelli et al., 2017; Prieto-Borja et al., 2017; Wang et al., 2017; Ye et al., 2017; Akhavan et al., 2018; Atefyekta et al., 2018; Ferraris et al., 2018; Hidalgo-Robatto et al., 2018; Pissinis et al., 2018; Santos-Coquillat et al., 2018; Zhang et al., 2018), *Porphyromonas gingivalis* (9.5%) (Gonçalves et al., 2010; Giordano et al., 2011; Holmberg et al., 2013; Eick et al., 2013; Roberts et al., 2013; Ciandrini et al., 2014; Hauser-Gerspach et al., 2014; Kang et al., 2014; Sahrmann et al., 2014; Schmidt et al., 2014; Jennings et al., 2015; Zhang et al., 2015; Chen et al., 2016; Batsukh et al., 2017; Kulkarni Aranya et al., 2017; Schmidt et al., 2017; Strever et al., 2017; Azizi et al., 2018; Lee et al., 2018; Vilarrasa et al., 2018; Huang et al., 2019), *Streptococcus sanguinis* (7.3%) (Duarte et al., 2009; Fröjd et al., 2011; Bürgers et al., 2012; Godoy-Gallardo et al., 2014; Hauser-Gerspach et al., 2014; Schmidt et al., 2014; Narendrakumar et al., 2015; Chen et al., 2016; Godoy-Gallardo et al., 2016; Guan et al., 2016; Kim et al., 2017; Ramesh et al., 2017; Hoyos-Nogués et al., 2018; Pantaroto et al., 2018; Souza et al., 2018), and *Streptococcus mutans* (7.3%) (Baffone et al., 2011; Giordano et al., 2011; Ntrouka et al., 2011; Roberts et al., 2013; Schmage et al., 2014; Ciandrini et al., 2014; Cruz et al., 2015; Narendrakumar et al., 2015; Yucesoy et al., 2015; Zhang et al., 2015; Ciandrini et al., 2016; Ye et al., 2017; Ciandrini et al., 2017; Ramesh et al., 2017; Fukushima et al., 2018; Huang et al., 2019).

### Cell Culture Studies

In addition, 37 of the included studies (*n* = 37, 31.9%) also performed cell culture in their methodology to study re-osseointegration (Schwarz et al., 2005; Ewald and Ihde, 2009; Giordano et al., 2011; Cortizo et al., 2012; Alcheikh et al., 2013; De Giglio et al., 2013; Eick et al., 2013; Holmberg et al., 2013; Godoy-Gallardo et al., 2014; Hauser-Gerspach et al., 2014; Duske et al., 2015; Janković et al., 2015; Lewandowska et al., 2015; Ayre et al., 2016; Cochis et al., 2016; Cotolan et al., 2016; Giannelli et al., 2016; Guan et al., 2016; Godoy-Gallardo et al., 2016; John et al., 2016; Kotsakis et al., 2016; Shi et al., 2016; Canullo et al., 2017; Cometa et al., 2017; Eick et al., 2017; Ferraris et al., 2017; Giannelli et al., 2017; Matthes et al., 2017; Ramesh et al., 2017; Wang et al., 2017; Ye et al., 2017; Zhang et al., 2017; Atefyekta et al., 2018; Hidalgo-Robatto et al., 2018; Hoyos-Nogués et al., 2018; Lee et al., 2018; Santos-Coquillat et al., 2018). The investigated cell types in this systematic review were osteogenic sarcoma (57.4%), epithelial (2.1%), fibroblast (17.0%), macrophages (4.3%), acute monocytic leukemia (2.1%), mesenchymal stem cells (4.3%), and stromal cells (2.1%).

This review has grouped the decontamination methods into two types: (i) biofilm-prevention method and (ii) biofilm-treatment method.

### Biofilm-Prevention Methods

The studies focused on the modification of titanium implant surfaces. For example, one of the included studies focused on the type of instrument used to modify the surfaces, either to smooth or to roughen them (Schmidt et al., 2017). This biofilm-preventing step occurs before the development of assumed biofilm. In addition to the surface roughness, materials and surface treatment, too, have an impact on the early attachment of bacteria (Batsukh et al., 2017). Therefore, it is essential to assess the effect of various implant surfaces available on the attachment of initial and late colonizing bacteria. In biofilm-treatment method, biofilm development has already taken place. Most of the included studies were for biofilm-treatment.

The most common preventive method of decontamination used in the included studies was the application of a coating on the titanium implant or discs. The mechanism of decontamination did not completely rely on the physical separation of the titanium alloy and oral microbiota.

### Biofilm-Treatment Methods

In a study using calcifying coating solutions (containing different ionic compositions of calcium, phosphate, and zinc), the degree of crystallinity was inversely proportional to antibacterial activity against *Porphyromonas gingivalis* in coated discs (Kulkarni Aranya et al., 2017). Lower the dissolution rate, lesser the antibacterial effect (Kulkarni Aranya et al., 2017). The addition of titanium dioxide nanoparticles boosted the antimicrobial efficacy of hydrogen peroxide solutions and delayed the redevelopment on surfaces which were previously contaminated by bacteria (Wiedmer et al., 2017).

The second most common biofilm-treatment method of decontamination in this review is by chemical treatment. However, similar to the previous findings, their results showed that not all of the agents used for disinfection in the clinic are effective in the removal of the biofilm. Some of the agents left behind live bacteria on the surface (Dostie et al., 2017). With regards to mechanical cleaning, a complete decontamination of a surface will not be possible as certain bacteria will continue to reside in “valleys and undercuts” (Schmage et al., 2014). In general, removal of greater than 96% biofilm was reported as satisfactory for clinical health, and this was achieved by the oscillating instruments and air polishing (Schmage et al., 2014). One study focused on the application of a direct contact ultrasound at low frequency (Granick et al., 2017). They described that the ultrasound biocidal activity occurred due to acoustic microstreaming and bubble cavitation, releasing considerable energy and damaging bacterial cell walls. In another study, implants were used as cathodes to generate an alkaline environment and reactive oxygen species, and showed a twofold reduction of bacteria compared with the untreated controls (Mohn et al., 2011). Mechanisms are similar to chemical treatments. For example, the ability of hydrogen peroxide to cause bacterial biofilm disruption is associated with oxidation of a number of cellular components and production of gas (Wiedmer et al., 2017).

*Staphylococcus aureus*, *Porphyromonas gingivalis*, *Streptococcus sanguinis*, and *Streptococcus mutans* are the commonly used oral microbiota for the study of dental biofilms reported in the included studies of this review. These bacteria are all responsible for peri-implantitis (Lavere et al., 2018). They can also exist in the oral saliva of healthy adults and later transform into opportunistic pathogens to become the main cause of the host diseases (Gao et al., 2018). *S. aureus* is a Gram-positive, aerobic cocci and may cause infections ranging from minor skin infections to pneumonia, bacteremia, and infective endocarditis (Oliveira et al., 2018). *P. gingivalis* is a Gram-negative anaerobic bacteria and has a strong positive correlation with periodontal diseases (Rafiei et al., 2017). *S. sanguinis*, previously known as *S. sanguis*, is a Gram-positive, non-spore-forming, facultative anaerobe and is typically found in a healthy plaque (Zhu et al., [[NoYear]]). *S. mutans* is a Gram-positive cocci, a facultative anaerobe responsible for the initiation of dental caries, which may lead to the formation of dental plaque and endocarditis (Daboor et al., 2015).

An important question for surface decontamination studies is whether the different patterns of biofilm play a clinical role (Trobos et al., 2018). Biofilm‐associated antimicrobial resistance is related to a number of factors. Established biofilms are resistant to antimicrobial agents as compared with planktonic bacteria which are attributed to biofilm properties, including nature and structure, poor antibiotic penetration, bacterial cells metabolic state, nutrient and oxygen availability, and antimicrobial resistance acquired by gene transfer and mutation (Arciola et al., 2018).

There are many other factors to consider if the decontamination method is successful or not. The first is the age of the biofilm. Half of the included studies in this review evaluated on biofilm aged less or equal to 24 h. However, this may also depend on the type of resident microbiota, maturity of the biofilm, and the number of bacterial cell clusters and micro colonies (Cao et al., 2018). This is a crucial factor especially for those treatment methods aimed at established biofilms, such as in clinically diagnosed peri-implantitis. Additionally, *in-vitro* developed biofilm is easily affected by surface configuration than biofilm that forms in the mouth (Bevilacqua et al., 2018). Nutrients and mixed bacterial species present in more favorable environment will encourage biofilm formation (Bevilacqua et al., 2018). Quantitative differences detected *in-vitro* amongst surfaces with dissimilar features might not predict the rate of *in-vivo* colonization. Various studies analyzed the efficacy of their decontamination methods using a scanning electron microscopy (SEM). However, the energy beam by SEM on its own can be a potential decontamination strategy as the high energy (in kV) can cause bacterial cell wall destruction and internal reversible changes to genetic materials (Ghomi et al., 2005). Therefore, when a high-energy SEM captured the images of bacterial matrix for quantification, their image analysis may introduce inaccuracies.

Re-osseointegration is also an important issue taken into account when decontamination methods are performed. About one-third of the included studies in this review investigated the cell adherence on the titanium implants with biofilm contamination. This phenomenon is described as “the race for the surface,” wherein bacterial and host cells compete in colonizing implant surface (Hoyos-Nogués et al., 2018). Previous systematic review on re-osseointegration has reported that the application of single decontamination measure was not sufficient to get a desirable treatment outcome for peri-implantitis (Madi et al., 2018). Therefore, further studies must be conducted to focus on the dual mechanism (or more) to encourage re-osseointegration after biofilm decontamination. So far, there is no “gold standard” that has been specified in the decontamination of dental implants. A decontamination treatment will need to be combined with mechanical therapy for a successful outcome (Prathapachandran and Suresh, 2012).

## Conclusion

In conclusion, this review found that the commonly used decontamination methods of microbial biofilm on dental implant surfaces focused on the addition of surface coating and chemical treatment. Further efforts should be aimed at finding the optimal implant surface property that features antimicrobial treatment that is effective and does not compromise osseointegration.

## Author Contributions

Conceptualization: JD, JK, RA. Methodology: JD, LM, SD, JK, RA. Software: JD, NR, LM, SD, JK, RA. Validation: JD, NR, JK, RA. Formal analysis: JD, NR, LM, SD, JK, RA. Investigation: JD, NR, LM, SD, JK, RA. Resources: JD, NR, LM, SD, JK, RA. Data curation: JD, NR, LM, SD, JK, RA. Writing—original draft preparation: JD, NR, SD, JK. Writing—review and editing: JD, NR, LM, SD, JK, RA. Supervision: JD, JK. Project administration: JD, NR, LM, SD, JK, RA. Funding acquisition: JD, JK, RA. All authors contributed to the article and approved the submitted version.

## Funding

The work was supported by the University Research Council grant under Universiti Brunei Darussalam (grant number: UBD/RSCH/URC/RG (b)/2018/004). Allied Grant-Universiti Brunei Darusslam for Publication Cost.

## Conflict of Interest

The authors declare that the research was conducted in the absence of any commercial or financial relationships that could be construed as a potential conflict of interest.

## Publisher’s Note

All claims expressed in this article are solely those of the authors and do not necessarily represent those of their affiliated organizations, or those of the publisher, the editors and the reviewers. Any product that may be evaluated in this article, or claim that may be made by its manufacturer, is not guaranteed or endorsed by the publisher.
