# Supplementary material for: Microbial Biofilm Decontamination on Dental Implant Surfaces: A Mini Review
Source: Front Cell Infect Microbiol. 2021 Oct 8;11:736186. doi: 10.3389/fcimb.2021.736186 (PMC8531646; doi:10.3389/fcimb.2021.736186)
Supplement: Supplementary file 1 [file DataSheet_2.docx]

**Supplementary Information 2. Coating of the titanium materials and the findings of the respective studies.**

| **No.** | **Method of decontamination** | **Study Findings** | **Type of decontamination method (Biofilm-Prevention or Biofilm-Treatment)** | **Reference** |
| --- | --- | --- | --- | --- |
| 1 | Nano-scale hydroxyapatite (nHA) and zinc oxide nanoparticles (nZnO) | Reduced numbers of facultatively anaerobic and *Streptococcus spp*. on all nano-coated surfaces were demonstrated. The proportion of non-viable microorganisms was shown to be higher on nZnO and composite (nZnO + nHA) coated surfaces compared with nHA coated and uncoated titanium. | Biofilm-Prevention | (Abdulkareem et al., 2015) |
| 2 | Calcium-phosphate based thin coatings | The coated surfaces inhibited the growth, colonization and adherence of *P. gingivalis*, resulted in reduced thickness of biofilms and bacterial inhibition in the culture medium as compared to the positive and negative controls. | Biofilm-Prevention | (Kulkarni Aranya et al., 2017) |
| 3 | Minocycline-loaded polyelectrolyte multilayers of hyaluronic acid (HA) and chitosan (CS) | Bacteria were more likely to colonize rough DMLS-Ti surfaces. Loading minocycline into HA/CS polyelectrolyte multilayers on DMLS-Ti surfaces primed with PTL can achieve antibacterial efficacy while retaining osteoblast functions. | Biofilm-Prevention | (Guan et al., 2016) |
| 4 | Lysophosphatidic acid (LPA) analogue (3S)1-fluoro-3-hydroxy-4-(oleoyloxy)butyl-1-phosphonate (FHBP) | FHBP-Ti did not allow the adherence of *S. aureus.* | Biofilm-Prevention | (Ayre et al., 2016) |
| 5 | Polytetrafluoroethylene (PTFE) coating | *S. oralis* attached and produced biofilm differently on different PTFE membrane types, and prevented *S. oralis* from crossing the barrier. | Biofilm-Prevention | (Trobos et al., 2018) |
| 6 | Multi-walled carbon nanotube (MWCNT), impregnated with Rifampicin | Titanium with coated surfaces caused a significant inhibition of biofilm formation for up to five days. | Biofilm-Prevention | (Hirschfeld et al., 2017) |
| 7 | Zirconium nitride (ZrN) coating | ZrN coating appears to rather modify the quantity of early bacterial adherence than the quality of the microbial community structure. | Biofilm-Prevention | (Größner-Schreiber et al., 2009) |
| 8 | Silver-coated titanium-aluminum-niobium (TiAlNb) | Silver coating of TiAlNb alloy produces a strong anti-staphylococcal effect and neither gamma sterilization nor alcohol disinfection influenced its outcome. | Biofilm-Prevention | (Kuehl et al., 2016) |
| 9 | Salt impregnation of implant materials | The resulting NaCl-coated surfaces showed good biocompatibility with osteoblast-like cells and resulted in a significant reduction of bacterial strains. | Biofilm-Prevention | (Ewald and Ihde, 2009) |
| 10 | Tantalum nitride (TaN)-decorated titanium | The biofilm experiment showed that the TaN-decorated Ti sample possessed good antibacterial performance. | Biofilm-Prevention | (Zhang et al., 2015) |
| 11 | Polyethylene glycol (PEG)-based anti-adhesive coating | The coated surfaces effectively inhibited the initial adhesion of *S. sanguinis.* | Biofilm-Prevention | (Hoyos-Nogués et al., 2018) |
| 12 | Triethoxysilypropyl succinic anhydride (TESPSA) silane | A noticeable reduction in the adhesion and early stages of biofilm formation of *S. sanguinis* and *L. salivarius* was observed. | Biofilm-Prevention | (Godoy-Gallardo et al., 2016) |
| 13 | Silver-doped hydroxyapatite thin films sputter | Less bacteria adhered to surfaces containing hydroxyapatite and silver. When the hydroxyapatite films were delaminated, silver ions were released which eventually killed bacteria in suspension. | Biofilm-Prevention | (Trujillo et al., 2012) |
| 14 | TiO_2_ films with different crystalline phases (anatase, rutile and a mixture of both) | Anatase and mixture-TiO_2_ showed antibacterial activity on oral bacterial biofilm. | Biofilm-Prevention | (Pantaroto et al., 2018) |
| 15 | Elastin-like polypeptide (ELP) surface coatings containing cell-adhesive peptide domains (RGD) using covalent chemistry | The coating with an antimicrobial peptide, RRPRPRPRPWWWW-NH2 (RRP9W4N), preserved its antibacterial activity up to 24 h. | Biofilm-Prevention | (Atefyekta et al., 2018) |
| 16 | Chitosan coatings loaded with silver-decorated calcium phosphate microspheres | Increasing concentrations of silver loaded on calcium phosphate microspheres within the chemically bound coating reduces bacterial viability by up to 90% in both anaerobic and aerobic pathogenic microorganisms. | Biofilm-Prevention | (Jennings et al., 2015) |
| 17 | Titanium/polybenzyl acrylate coating | The coating is inhibited the adhesion of early colonizers on titanium surfaces. | Biofilm-Prevention | (Cortizo et al., 2012) |
| 18 | DNase I coating of titanium | The coating showed significant effects in preventing the adhesion of *Streptococcus mutans* and *Staphylococcus aureus,* inhibiting biofilm formation over a time span of 24 h. | Biofilm-Prevention | (Ye et al., 2017) |
| 19 | Fluor‑carbonated hydroxyapatite coatings | Fluorine promoted a significant reduction in biofilm formation by *Staphylococcus aureus* and *Staphylococcus epidermidis.* | Biofilm-Prevention | (Hidalgo-Robatto et al., 2018) |
| 20 | Minocycline-loaded chitosan/alginate multilayer | Minocycline killed planktonic and adherent bacteria. The surface charge and hydrophilicity of the coatings and antibacterial ability of chitosan itself played important roles in the antibacterial performance. | Biofilm-Prevention | (Lv et al., 2014) |
| 21 | Nanogrooves and keratin nanofibers | Neither nanogrooves nor keratin nanofibers increase bacterial biofilm adhesion in comparison with mirror polished surfaces. | Biofilm-Prevention | (Ferraris et al., 2017) |
| 22 | Sol-gel synthesis of titanium dioxide and Ag-TiO2 coatings | The adhesion of *Pseudomonas aeruginosa* was reduced in the presence of silver and the bacteria shape destroyed. | Biofilm-Prevention | (Cotolan et al., 2016) |
| 23 | Modified oxide layer with embedded silver nanoparticles | 33% reduction in the staphylococcal adhesion can be reached on the chemically treated surface without introduction of silver ions in the process with respect to the polished surface. These result evidences the ability of Ti6Al4V surfaces with a surface nanotexture to limit bacterial adhesion, compared to a mirror polished control, even without the addition of silver. | Biofilm-Prevention | (Ferraris et al., 2018) |
| 24 | Composite coating on highly ordered nanoporous silica and silver nanoparticles | AgNP/NSC coating produces a strong antibacterial effect on the titanium surface by killing the adherent bacteria and inhibiting biofilm formation. | Biofilm-Prevention | (Massa et al., 2014) |
| 25 | Plasma electrolytic oxidation (PEO) in two different electrolytes; a conventional Ca/P-based electrolyte and its modified version with added F and Si | The coating with the greatest content of fluoride demonstrated the most pronounced antibacterial effect with respect to S. aureus reducing the surface coverage down to 60% and leading to death of 3% of bacteria. | Biofilm-Prevention | (Santos-Coquillat et al., 2018) |
| 26 | Silver or gallium doping | Gallium or silver can provide antibacterial properties and maintain high osseo-integrative potential. | Biofilm-Prevention | (Cochis et al., 2016) |
| 27 | Silver nanoparticles (AgNPs) and/or ampicillin (AMP) | Ti-AMP and Ti-AgNPs-AMP showed the highest antibacterial effect, indicating that the -lactam ring functionality is maintained after the adsorption process. Moreover, the antimicrobial capacity is maintained over time due to a two-pathway antibacterial action mechanism: death by contact (AMP) and death by release (AgNPs). The effect of AMP prevails on AgNPs at early stages of bacterial adhesion, while AgNPs are responsible for sustaining the relatively low but steady release of silver. | Biofilm-Prevention | (Pissinis et al., 2018) |
| 28 | Polyacrylate-based hydrogel thin coatings containing poly(ethylene glycol diacrylate)–co–acrylic acid (PEGDA-AA) | AgNP-modified PEGDA-AA coatings showed a good antibacterial activity against *S. aureus, P. aeruginosa,* *E. coli* and two clinical *S. aureus* cultures. | Biofilm-Prevention | (De Giglio et al., 2013) |
| 29 | Silver and copper | The Cu alloy had moderate antibacterial properties. The addition of Ag appeared to enhance more antibacterial properties, possibly as a result of the low alloying content. | Biofilm-Prevention | (Macpherson et al., 2017) |
| 30 | Chlorhexidine hexametaphosphate nanoparticles | CHX HMP NP-coated surfaces exhibited antimicrobial efficacy against *Streptococcus gordonii* within 8 h. The antimicrobial efficacy was greater in the presence of an acquired pellicle which is postulated to be due to retention of soluble CHX by the pellicle. | Biofilm-Prevention | (Wood et al., 2015) |
| 31 | Nanoporous titanium dioxide coating with anodized Ca2+ modification | Nano-topographical modification of smooth titanium surfaces did not cause significantly greater adhesion and biofilm formation by *S. sanguinis* and *A. naeslundii.* *in-vitro* than was found on turned surfaces or those treated with Ca^2+^ incorporation during anodic oxidation. | Biofilm-Prevention | (Fröjd et al., 2011) |
| 32 | Mg-ion and Ca-ion | adhesions of *P. gingivalis* and *F. nucleatum* on the ion-implanted surfaces were significantly larger and stronger than on control surfaces. The effect by ion type was subtle. | Biofilm-Prevention | (Kang et al., 2014) |
| 33 | Electrophoretic deposition of graphene oxide reinforced chitosan–hydroxyapatite nanocomposite coatings | The largest number of *S. aureus* cells with round shape morphology were observed on the HA coating. In contrast, the number of adherent bacterial cells decreased remarkably on the CS–HA and 1.7 GO–CS–HA coatings, which was consistent with the result of the bactericidal CS–graphene oxide layers. | Biofilm-Prevention | (Shi et al., 2016) |
| 34 | Fluorophosphonate-functionalised titanium via a pre-adsorbed alkane phosphonic acid | Of further significance are the findings of an anti-adherent property of FHBP-Ti towards *S. aureus*, a clinically relevant bacterial species associated with sepsis-induced implant failures. | Biofilm-Prevention | (Ayre et al., 2016) |
| 35 | Novaron (grade VZ 600; Toagosei, Tokyo, Japan), a commercially available inorganic antimicrobial powder made from glass, with the functional material being zinc: ZnO (20%–30%)- P2O5-SiO2-Al2O3-CaO | Compared with the initial number of bacteria, the number of *S. aureus* was decreased almost 10-fold on control samples following a 24-h incubation, but more than 20 000-fold on coated samples (*p* = 0.007); the degree of antimicrobial activity, calculated as described earlier, was more than 3.3. The number of *P. aeruginosa* increased almost fourfold on control samples following a 24-h incubation but decreased more than 15 000-fold on coated samples (*p* = 0.035); the degree of antimicrobial activity was more than 4.9. Finally, *K. pneumoniae* increased almost sevenfold on control samples following a 24-h incubation but decreased almost eightfold on coated samples (*p* = 0.019); the degree of antimicrobial activity was 1.8. | Biofilm-Prevention | (Tamai et al., 2009) |
| 36 | antibiotic-loaded poly(3-hydroxybutyrate) (PHB) nano- and micro-spheres and poly(ethylene glycol) (PEG) as an antifouling agent | Ti surfaces treated with 3-chloropropyltriethoxysilane (CPTES) and poly(ethylene glycol) (PEG) show a significant decrease on the bacterial counting. The synergistic effect of the PEG together with Doxy-loaded PHB spheres reduces bacterial adhesion (*Escherichia coli* and *Staphylococcus aureus*). | Biofilm-Prevention | (Rodríguez-Contreras et al., 2016) |
| 37 | Osteogenic micro-arc oxidation (MAO) coatings with micro/nanoscale porous topography with immobilized heparin and vancomycin | Appreciable antibacterial and antibiofilm performances were achieved against *S. aureus.* | Biofilm-Prevention | (Zhang et al., 2018) |
| 38 | PolyNaSS grafting | PolyNaSS grafting on cpTi decreased the number of adherent *S. aureus*, did not compromise functions such as adhesion and proliferation and promoted differentiation of the bone-forming cells. | Biofilm-Prevention | (Alcheikh et al., 2013) |
| 39 | Silver-loaded chitosan coating | Silver loading of approximately 5 μg/cm^2^ is enough to kill planktonic and adherent bacteria during the first stages of implantation, while keeping cytocompatibility at a suitable level. | Biofilm-Prevention | (Cometa et al., 2017) |
| 40 | Lignin addition to simple and silver-doped hydroxyapatite thin films synthesized by matrix-assisted pulsed laser evaporation | The coated composite secured a prolonged release of silver ions, being protective both against the initial phase of microbial colonization and the mature biofilm development. The lignin addition boosted the anti-microbial activity of HA doped with silver ions against both bacterial and fungal biofilms. | Biofilm-Prevention | (Janković et al., 2015) |
| 41 | TiN, TiO2 single layer, and TiN/TiO2 multilayer coatings | The coated substrates could help to effectively reduce the bacterial adhesion and biofilm formations. | Biofilm-Prevention | (Kaliaraj et al., 2014) |
| 42 | Magnetron sputtered TiN/VN nanoscale multilayers | The attached bacteria were higher on uncoated substrates with higher roughness than on coated substrates. | Biofilm-Prevention | (Subramanian et al., 2012) |
| 43 | Antibacterial coatings: silver nanoparticles and TESPSA silanization | TESPSA reduced cellular viability and bacterial adhesion in a multispecies *in-vitro* biofilm. However, silver nanoparticles were not able to confer these antibacterial properties. | Biofilm-Prevention | (Vilarrasa et al., 2018) |
| 44 | highly ordered titanium dioxide nanotube coatings | Some types of titanium dioxide nanotube covers were able to reduce staphylococcal aggregates/ biofilm formation | Biofilm-Prevention | (Lewandowska et al., 2015) |
| 45 | Zirconium nitride (ZrN) coating | Zirconium-nitride coating of a Ti surface altered the microbial composition (24 hours), with augmentation of *Lactobacillus*-related phylotypes later. Long term exposure (14 days) of dental implant surfaces to microbes resulted in a significantly different composition of the biofilm on all three tested surfaces. | Biofilm-Prevention | (Rehman et al., 2012) |
| 46 | A novel antibiotic nanodelivery system based on self-decomposable silica-gentamycin (SG) nanoparticles, fabricated using an innovative one-pot solution | SG nanoparticles from the antibacterial titanium coating continuously released gentamycin and inhibited *S. aureus* growth. | Biofilm-Treatment | (Wang et al., 2017) |
| 47 | Dimethylaminododecyl methacrylate (DMADDM), a novel quaternary ammonium salts (QAS) | The DMADDM modified dental implant not only inhibited the biomass accumulation and metabiotic activity of saliva-derived biofilm, but also regulated microbial ecosystem to healthier condition. It was found that 5 mg/mL DMADDM in coating solution could provide ideal anti-bacterial and microecosystem-regulating capabilities of titanium implants. | Biofilm-Treatment | (Li et al., 2017) |
| 48 | Antimicrobial peptide GL13K, developed from the human salivary protein BPIFA2 | The covalently-immobilized GL13K antimicrobial peptide coating had excellent antimicrobial activity when exposed to *S. gordonii* cultures in a drip flow biofilm reactor system. These culturing conditions in combination with the activity of the GL13K peptide coatings resulted in rupture of the cell wall of Gram positive bacteria. | Biofilm-Treatment | (Chen et al., 2014) |
| 49 | Biosurfactants produced by *Lactobacillus reuteri* DSM17938, *Lactobacillus acidophilus* DDS-1, *Lactobacillus rhamnosus* ATCC 53103, and *Lactobacillus paracasei* B21060 | Biosurfactants of LAB origin reduces the ability of streptococci to adhere and develop biofilm on oral surfaces. | Biofilm-Treatment | (Ciandrini et al., 2016) |
| 50 | Nanostructured crystalline titaniumdioxide coatings deposited by cathodic arc | 90 % reduction of viable bacteria was achieved in only 2 min with a UV dose of 2.4 J. | Biofilm-Treatment and Biofilm-Prevention | (Lilja et al., 2012) |

**References**

Abdulkareem, E. H., Memarzadeh, K., Allaker, R. P., Huang, J., Pratten, J., and Spratt, D. (2015). Anti-biofilm activity of zinc oxide and hydroxyapatite nanoparticles as dental implant coating materials. *J. Dent.* 43, 1462–1469. doi:10.1016/j.jdent.2015.10.010.

Alcheikh, A., Pavon-Djavid, G., Helary, G., Petite, H., Migonney, V., and Anagnostou, F. (2013). PolyNaSS grafting on titanium surfaces enhances osteoblast differentiation and inhibits Staphylococcus aureus adhesion. *J. Mater. Sci. Mater. Med.* 24, 1745–1754. doi:10.1007/s10856-013-4932-3.

Atefyekta, S., Pihl, M., Lindsay, C., Heilshorn, S. C., and Andersson, M. (2018). Antibiofilm elastin-like polypeptide coatings: functionality, stability, and selectivity. *Acta Biomater.* 83, 245–256. doi:10.1016/j.actbio.2018.10.039.

Ayre, W. N., Scott, T., Hallam, K., Blom, A. W., Denyer, S., Bone, H. K., et al. (2016). Fluorophosphonate-functionalised titanium via a pre-adsorbed alkane phosphonic acid: a novel dual action surface finish for bone regenerative applications. *J. Mater. Sci. Mater. Med.* 27, 1–12. doi:10.1007/s10856-015-5644-7.

Chen, X., Hirt, H., Li, Y., Gorr, S. U., and Aparicio, C. (2014). Antimicrobial GL13K peptide coatings killed and ruptured the wall of streptococcus gordonii and prevented formation and growth of biofilms. *PLoS One* 9. doi:10.1371/journal.pone.0111579.

Ciandrini, E., Campana, R., Casettari, L., Perinelli, D. R., Fagioli, L., Manti, A., et al. (2016). Characterization of biosurfactants produced by Lactobacillus spp. and their activity against oral streptococci biofilm. *Appl. Microbiol. Biotechnol.* 100, 6767–6777. doi:10.1007/s00253-016-7531-7.

Cochis, A., Azzimonti, B., Della Valle, C., De Giglio, E., Bloise, N., Visai, L., et al. (2016). The effect of silver or gallium doped titanium against the multidrug resistant Acinetobacter baumannii. *Biomaterials* 80, 80–95. doi:10.1016/j.biomaterials.2015.11.042.

Cometa, S., Bonifacio, M. A., Baruzzi, F., de Candia, S., Giangregorio, M. M., Giannossa, L. C., et al. (2017). Silver-loaded chitosan coating as an integrated approach to face titanium implant-associated infections: analytical characterization and biological activity. *Anal. Bioanal. Chem.* 409, 7211–7221. doi:10.1007/s00216-017-0685-z.

Cortizo, M. C., Oberti, T. G., Cortizo, M. S., Cortizo, A. M., and Fernández Lorenzo De Mele, M. A. (2012). Chlorhexidine delivery system from titanium/polybenzyl acrylate coating: Evaluation of cytotoxicity and early bacterial adhesion. *J. Dent.* 40, 329–337. doi:10.1016/j.jdent.2012.01.008.

Cotolan, N., Rak, M., Bele, M., Cör, A., Muresan, L. M., and Milošev, I. (2016). Sol-gel synthesis, characterization and properties of TiO2and Ag-TiO2coatings on titanium substrate. *Surf. Coatings Technol.* 307, 790–799. doi:10.1016/j.surfcoat.2016.09.082.

De Giglio, E., Cafagna, D., Cometa, S., Allegretta, A., Pedico, A., Giannossa, L. C., et al. (2013). An innovative, easily fabricated, silver nanoparticle-based titanium implant coating: Development and analytical characterization. *Anal. Bioanal. Chem.* 405, 805–816. doi:10.1007/s00216-012-6293-z.

Ewald, A., and Ihde, S. (2009). Salt impregnation of implant materials. *Oral Surgery, Oral Med. Oral Pathol. Oral Radiol. Endodontology* 107, 790–795. doi:10.1016/j.tripleo.2008.12.028.

Ferraris, S., Spriano, S., Miola, M., Bertone, E., Allizond, V., Cuffini, A. M., et al. (2018). Surface modification of titanium surfaces through a modified oxide layer and embedded silver nanoparticles: Effect of reducing/stabilizing agents on precipitation and properties of the nanoparticles. *Surf. Coatings Technol.* 344, 177–189. doi:10.1016/j.surfcoat.2018.03.020.

Ferraris, S., Truffa Giachet, F., Miola, M., Bertone, E., Varesano, A., Vineis, C., et al. (2017). Nanogrooves and keratin nanofibers on titanium surfaces aimed at driving gingival fibroblasts alignment and proliferation without increasing bacterial adhesion. *Mater. Sci. Eng. C* 76, 1–12. doi:10.1016/j.msec.2017.02.152.

Fröjd, V., Linderbäck, P., Wennerberg, A., Chávez de Paz, L., Svensäter, G., and Davies, J. R. (2011). Effect of nanoporous TiO2 coating and anodized Ca2+ modification of titanium surfaces on early microbial biofilm formation. *BMC Oral Health* 11, 8. doi:10.1186/1472-6831-11-8.

Godoy-Gallardo, M., Guillem-Marti, J., Sevilla, P., Manero, J. M., Gil, F. J., and Rodriguez, D. (2016). Anhydride-functional silane immobilized onto titanium surfaces induces osteoblast cell differentiation and reduces bacterial adhesion and biofilm formation. *Mater. Sci. Eng. C* 59, 524–532. doi:10.1016/j.msec.2015.10.051.

Größner-Schreiber, B., Teichmann, J., Hannig, M., Dörfer, C., Wenderoth, D. F., and Ott, S. J. (2009). Modified implant surfaces show different biofilm compositions under in vivo conditions. *Clin. Oral Implants Res.* 20, 817–826. doi:10.1111/j.1600-0501.2009.01729.x.

Guan, B., Wang, H., Xu, R., Zheng, G., Yang, J., Liu, Z., et al. (2016). Establishing Antibacterial Multilayer Films on the Surface of Direct Metal Laser Sintered Titanium Primed with Phase-Transited Lysozyme. *Sci. Rep.* 6, 1–12. doi:10.1038/srep36408.

Hidalgo-Robatto, B. M., Aguilera-Correa, J. J., López-Álvarez, M., Romera, D., Esteban, J., González, P., et al. (2018). Fluor‑carbonated hydroxyapatite coatings by pulsed laser deposition to promote cell viability and antibacterial properties. *Surf. Coatings Technol.* 349, 736–744. doi:10.1016/j.surfcoat.2018.06.047.

Hirschfeld, J., Akinoglu, E. M., Wirtz, D. C., Hoerauf, A., Bekeredjian-Ding, I., Jepsen, S., et al. (2017). Long-term release of antibiotics by carbon nanotube-coated titanium alloy surfaces diminish biofilm formation by Staphylococcus epidermidis. *Nanomedicine Nanotechnology, Biol. Med.* 13, 1587–1593. doi:10.1016/j.nano.2017.01.002.

Hoyos-Nogués, M., Buxadera-Palomero, J., Ginebra, M. P., Manero, J. M., Gil, F. J., and Mas-Moruno, C. (2018). All-in-one trifunctional strategy: A cell adhesive, bacteriostatic and bactericidal coating for titanium implants. *Colloids Surfaces B Biointerfaces* 169, 30–40. doi:10.1016/j.colsurfb.2018.04.050.

Janković, A., Eraković, S., Ristoscu, C., Mihailescu (Serban), N., Duta, L., Visan, A., et al. (2015). Structural and biological evaluation of lignin addition to simple and silver-doped hydroxyapatite thin films synthesized by matrix-assisted pulsed laser evaporation. *J. Mater. Sci. Mater. Med.* 26. doi:10.1007/s10856-014-5333-y.

Jennings, J. A., Velasquez Pulgarin, D. A., Kunwar, D. L., Babu, J., Mishra, S., and Bumgardner, J. (2015). Bacterial inhibition by chitosan coatings loaded with silver-decorated calcium phosphate microspheres. *Thin Solid Films* 596, 83–86. doi:10.1016/j.tsf.2015.08.060.

Kaliaraj, G. S., Ramadoss, A., Sundaram, M., Balasubramanian, S., and Muthirulandi, J. (2014). Studies of calcium-precipitating oral bacterial adhesion on TiN, TiO2single layer, and TiN/TiO2multilayer-coated 316L SS. *J. Mater. Sci.* 49, 7172–7180. doi:10.1007/s10853-014-8425-7.

Kang, S. N., Jeong, C. M., Jeon, Y. C., Byon, E. S., Jeong, Y. S., and Cho, L. R. (2014). Effects of Mg-ion and Ca-ion implantations on P. gingivalis and F. nucleatum adhesion. *Tissue Eng. Regen. Med.* 11, 39–46. doi:10.1007/s13770-013-1104-y.

Kuehl, R., Brunetto, P. S., Woischnig, A.-K., Varisco, M., Rajacic, Z., Vosbeck, J., et al. (2016). Preventing Implant-Associated Infections by Silver Coating. *Antimicrob. Agents Chemother.* 60, 2467–2475. doi:10.1128/AAC.02934-15.

Kulkarni Aranya, A., Pushalkar, S., Zhao, M., LeGeros, R. Z., Zhang, Y., and Saxena, D. (2017). Antibacterial and bioactive coatings on titanium implant surfaces. *J. Biomed. Mater. Res. - Part A* 105, 2218–2227. doi:10.1002/jbm.a.36081.

Lewandowska, Ż., Piszczek, P., Radtke, A., Jędrzejewski, T., Kozak, W., and Sadowska, B. (2015). The evaluation of the impact of titania nanotube covers morphology and crystal phase on their biological properties. *J. Mater. Sci. Mater. Med.* 26, 1–12. doi:10.1007/s10856-015-5495-2.

Li, B., Ge, Y., Wu, Y., Chen, J., Xu, H. H. K., Yang, M., et al. (2017). Anti-bacterial and microecosystem-regulating effects of dental implant coated with dimethylaminododecyl methacrylate. *Molecules* 22, 1–11. doi:10.3390/molecules22112009.

Lilja, M., Forsgren, J., Welch, K., Åstrand, M., Engqvist, H., and Strømme, M. (2012). Photocatalytic and antimicrobial properties of surgical implant coatings of titanium dioxide deposited though cathodic arc evaporation. *Biotechnol. Lett.* 34, 2299–2305. doi:10.1007/s10529-012-1040-2.

Lv, H., Chen, Z., Yang, X., Cen, L., Zhang, X., and Gao, P. (2014). Layer-by-layer self-assembly of minocycline-loaded chitosan/alginate multilayer on titanium substrates to inhibit biofilm formation. *J. Dent.* 42, 1464–1472. doi:10.1016/j.jdent.2014.06.003.

Macpherson, A., Li, X., McCormick, P., Ren, L., Yang, K., and Sercombe, T. B. (2017). Antibacterial Titanium Produced Using Selective Laser Melting. *Jom* 69, 2719–2724. doi:10.1007/s11837-017-2589-y.

Massa, M. A., Covarrubias, C., Bittner, M., Fuentevilla, I. A., Capetillo, P., Von Marttens, A., et al. (2014). Synthesis of new antibacterial composite coating for titanium based on highly ordered nanoporous silica and silver nanoparticles. *Mater. Sci. Eng. C* 45, 146–153. doi:10.1016/j.msec.2014.08.057.

Pantaroto, H. N., Ricomini-Filho, A. P., Bertolini, M. M., Dias da Silva, J. H., Azevedo Neto, N. F., Sukotjo, C., et al. (2018). Antibacterial photocatalytic activity of different crystalline TiO2phases in oral multispecies biofilm. *Dent. Mater.* 34, e182–e195. doi:10.1016/j.dental.2018.03.011.

Pissinis, D. E., Benítez, G. A., and Schilardi, P. L. (2018). Two-step biocompatible surface functionalization for two-pathway antimicrobial action against Gram-positive bacteria. *Colloids Surfaces B Biointerfaces* 164, 262–271. doi:10.1016/j.colsurfb.2018.01.057.

Rehman, A., Hu, J., Ott, S. J., and Grössner-Schreiber, B. (2012). Microbial community composition on modified dental implant surfaces: an in vivo study. *Int. J. Oral Maxillofac. Implants* 27, 811–9.

Rodríguez-Contreras, A., Marqués-Calvo, M. S., Gil, F. J., and Manero, J. M. (2016). Modification of titanium surfaces by adding antibiotic-loaded PHB spheres and PEG for biomedical applications. *J. Mater. Sci. Mater. Med.* 27. doi:10.1007/s10856-016-5723-4.

Santos-Coquillat, A., Gonzalez Tenorio, R., Mohedano, M., Martinez-Campos, E., Arrabal, R., and Matykina, E. (2018). Tailoring of antibacterial and osteogenic properties of Ti6Al4V by plasma electrolytic oxidation. *Appl. Surf. Sci.* 454, 157–172. doi:10.1016/j.apsusc.2018.04.267.

Shi, Y. Y., Li, M., Liu, Q., Jia, Z. J., Xu, X. C., Cheng, Y., et al. (2016). Electrophoretic deposition of graphene oxide reinforced chitosan–hydroxyapatite nanocomposite coatings on Ti substrate. *J. Mater. Sci. Mater. Med.* 27, 1–13. doi:10.1007/s10856-015-5634-9.

Subramanian, B., Ananthakumar, R., Kobayashi, A., and Jayachandran, M. (2012). Surface modification of 316L stainless steel with magnetron sputtered TiN/VN nanoscale multilayers for bio implant applications. *J. Mater. Sci. Mater. Med.* 23, 329–338. doi:10.1007/s10856-011-4500-7.

Tamai, K., Kawate, K., Kawahara, I., Takakura, Y., and Sakaki, K. (2009). Inorganic antimicrobial coating for titanium alloy and its effect on bacteria. *J. Orthop. Sci.* 14, 204–209. doi:10.1007/s00776-008-1306-7.

Trobos, M., Juhlin, A., Shah, F. A., Hoffman, M., Sahlin, H., and Dahlin, C. (2018). In vitro evaluation of barrier function against oral bacteria of dense and expanded polytetrafluoroethylene (PTFE) membranes for guided bone regeneration. *Clin. Implant Dent. Relat. Res.* 20, 738–748. doi:10.1111/cid.12629.

Trujillo, N. A., Oldinski, R. A., Ma, H., Bryers, J. D., Williams, J. D., and Popat, K. C. (2012). Antibacterial effects of silver-doped hydroxyapatite thin films sputter deposited on titanium. *Mater. Sci. Eng. C* 32, 2135–2144. doi:10.1016/j.msec.2012.05.012.

Vilarrasa, J., Delgado, L. M., Galofré, M., Àlvarez, G., Violant, D., Manero, J. M., et al. (2018). In vitro evaluation of a multispecies oral biofilm over antibacterial coated titanium surfaces. *J. Mater. Sci. Mater. Med.* 29. doi:10.1007/s10856-018-6168-8.

Wang, J., Wu, G., Liu, X., Sun, G., Li, D., and Wei, H. (2017). A decomposable silica-based antibacterial coating for percutaneous titanium implant. *Int. J. Nanomedicine* 12, 371–379. doi:10.2147/IJN.S123622.

Wood, N. J., Jenkinson, H. F., Davis, S. A., Mann, S., O’Sullivan, D. J., and Barbour, M. E. (2015). Chlorhexidine hexametaphosphate nanoparticles as a novel antimicrobial coating for dental implants. *J. Mater. Sci. Mater. Med.* 26, 1–10. doi:10.1007/s10856-015-5532-1.

Ye, J., Shao, C., Zhang, X., Guo, X., Gao, P., Cen, Y., et al. (2017). Effects of DNase I coating of titanium on bacteria adhesion and biofilm formation. *Mater. Sci. Eng. C* 78, 738–747. doi:10.1016/j.msec.2017.04.078.

Zhang, T., Zhou, W., Jia, Z., Wei, Q., Fan, D., Yan, J., et al. (2018). Polydopamine-assisted functionalization of heparin and vancomycin onto microarc-oxidized 3D printed porous Ti6Al4V for improved hemocompatibility, osteogenic and anti-infection potencies. *Sci. China Mater.* 61, 579–592. doi:10.1007/s40843-017-9208-x.

Zhang, Y., Zheng, Y., Li, Y., Wang, L., Bai, Y., Zhao, Q., et al. (2015). Tantalum nitride-decorated titanium with enhanced resistance to microbiologically induced corrosion and mechanical property for dental application. *PLoS One* 10, 1–22. doi:10.1371/journal.pone.0130774.
