# Supplementary material for: Microbial Biofilm Decontamination on Dental Implant Surfaces: A Mini Review
Source: Front Cell Infect Microbiol. 2021 Oct 8;11:736186. doi: 10.3389/fcimb.2021.736186 (PMC8531646; doi:10.3389/fcimb.2021.736186)
Supplement: Supplementary file 2 [file DataSheet_3.docx]

**MINI REVIEW**

**Front. Cell. Infect. Microbiol. | doi: 10.3389/fcimb.2021.736186**

**Dhaliwal, J.S. et al**

**Microbial biofilm decontamination on titanium implant surfaces: A mini review**

**Supplementary Information 3. Decontamination methods using antimicrobial drugs, chemical treatment, electrochemical treatment, probiotic and the findings of the respective studies.**

| **Type of method** | **Method of decontamination** | **Study Findings** | **Type of decontamination method (Biofilm-Prevention or Biofilm Treatment)** | **Reference** |
| --- | --- | --- | --- | --- |
| Antimicrobial drugs | Fluconazole (Hospira, Lake Forest, IL, USA), amphotericin B (Gibco Life Technologies, Grand Island, NY, USA), caspofungin (Merck & Co., Inc., Whitehouse Station, NJ, USA), and clindamycin (RPI Corp., Prospect, IL, USA) | Mixed biofilms formed on titanium discs showed a high level of resistance to combination therapy with antifungal and antibacterial drugs. | Biofilm-Treatment | (Montelongo-Jauregui et al., 2018) |
| Argon treatment | Argon atmospheric pressure dielectric barrier discharge (APDBD) | Bacterial adhesion was significantly reduced even after 60 seconds of argon treatment. | Biofilm-Treatment | (Canullo et al., 2017) |
| Chemical | Oligosaccharide nanomedicine (OligoG) | OligoG and triclosan when used in combination produced an enhanced antimicrobial effect against two important oral pathogens and reduced bacterial attachment to dental materials such as titanium, even at reduced triclosan concentrations. | Biofilm-Prevention | (Roberts et al., 2013) |
| Chemical | Aqueous solutions of chlorhexidine digluconate (CHX), obtained as a 20% (w/v) solution (Sigma, Milano, Italy), Curasept (Curaden Healthcare, Saronno, Italy) containing 0.2% CHX; Listerine (Pfizer, Latina, Italy) containing antibacterial essential oils (mint, thymus, eucalypt, birch), Meridol (GABA Vebas s.r.l., Roma, Italy) with a particular combination of aminefluoride/stannous fluoride, Buccagel (Curaden Healthcare) containing hexetidine, methylparaben and propilparaben | The tested mouthrinses, compared to reference CHX 0.2%, have demonstrated a significantly lower antibacterial activity than Listerine towards the experimental biofilms. | Biofilm-Prevention | (Baffone et al., 2011) |
| Chemical | An implant-paste, developed by combining a thickening agent made of 2D nanocrystalline magnesium phosphate (NMP) inorganic gel and hydrated silica nanoparticles (Sigma-Aldrich, Oakville, Canada) (containing 20, 30, 50, and 60% nanoparticles) | The implant-paste significantly cleans biofilm contaminated Ti without damaging its surface integrity compared to the rotary brush alone, or the brush combined with a commercial toothpaste. | Biofilm-Treatment | (Al-Hashedi et al., 2018) |
| Chemical | Hydrogen peroxide titanium dioxide (H_2_O_2_–TiO_2_) suspension | The addition of TiO_2_ nanoparticles enhanced the antibacterial effect of H_2_O_2_. | Biofilm-Treatment | (Wiedmer et al., 2017) |
| Chemical | Clinically available and commonly used chemotherapeutic agents (ChAs): 0.12% chlorhexidine, 20% citric acid (CA), 24%-EDTA/1.5%-sodium hypochlorite (NaOCl-EDTA), or sterile saline (SS) | Bacterial counts after decontamination confirmed that all of the ChAs were antimicrobial. The antimicrobial effect of the tested ChAs was greater for the CA and NaOCl-EDTA groups, followed by the CHX group, as compared with the contaminated controls. SS only had minimal antimicrobial efficacy. | Biofilm-Treatment | (Kotsakis et al., 2016) |
| Chemical | 0.12% chlorhexidine (CHX) (Periogard- Colgate-Palmolive Company, São Paulo, Brazil), chloramine T (CHT) (Trihydral - Perland Pharmacos Ltda., Londrina, Brazil), triclosan (Plax - Colgate-Palmolive Company, São Paulo, Brazil), and essential oils (EO) containing eucalyptol, thymol, methyl salicylate, and menthol (Listerine - Johnson & Johnson do Brasil Ind. e Com. de Produtos para Saúde Ltda., São Paulo, Brazil) | Different antiseptics reduced the number of bacteria in titanium implants. However, CHX was not as good against microcosm biofilm formation as CHT and EO which were efficient in reducing the biofilm biomass compared with saline solution. | Biofilm-Treatment | (Verardi et al., 2016) |
| Chemical | Tetracycline paste, 1% chlorhexidine gel (CHX), 35% phosphoric acid gel (Etch) or a novel chemical formula (0.3% cetrimide, 0.1% CHX and 0.5% EDTA) | Rinsing with saline was effective in removing the majority of the biofilm. None of the chemicals tested were superior to saline rinsing. | Biofilm-Treatment | (Dostie et al., 2017) |
| Chemical | Citric acid | Citric acid showed a significant reduction (≈ 5-log reduction) in the biofilm formed in situ compared with the control group. The acid treatment did not favor the recolonization of bacteria. | Biofilm-Treatment | (Souza et al., 2018) |
| Chemical | Hypochlorous acid (HOCl), sodium hypochlorite (NaOCl) and chlorhexidine (CHX) | HOCl is effective for cleaning biofilm-contaminated implant surfaces. The efficacy of HOCl is equivalent to NaOCl and CHX. The concentration of HOCl (0.018%) was lower than those for NaOCl (1.3%) and CHX (0.2%) but still showed efficacy against the tested bacterial species. | Biofilm-Treatment | (Chen et al., 2016) |
| Chemical | Cinnamon oil and clove oil (Abbey Essentials) | The tested natural oils were shown to be effective in inhibiting biofilm formation on all tested dental implant materials. | Biofilm-Treatment | (Diab Al-Radha et al., 2013) |
| Chemical | Citric acid (CA), Ardox-X, hydrogen peroxide (H_2_O_2_), chlorhexidine (CHX) and water | CA demonstrated the greatest decontamination capacity with respect to both the killing and the removal of biofilm cells. | Biofilm-Treatment | (Ntrouka et al., 2011) |
| Chemical | Sodium fluoride (NaF) | Fluoride can inhibit bacterial acid production in the presence of glucose. | Biofilm-Treatment | (Fukushima et al., 2018) |
| Chemical | 1% sodium hypochlorite, 3% hydrogen peroxide, 0.2% chlorhexidine gluconate, 40% citric acid, Plax, or Listerine | Sodium hypochlorite was effective against all tested microbial species, whereas hydrogen peroxide was solely effective against *C. albicans*. CHX, Listerine, citric acid and Plax showed antimicrobial activity against *S. sanguinis* and *C. albicans*. | Biofilm-Treatment | (Bürgers et al., 2012) |
| Chemical | Fluoride | Fluoride interaction with biofilms can significantly influence the tribological properties of commercially pure titanium in the oral cavity. | Biofilm-Treatment | (Cruz et al., 2015) |
| Chemical | Rubbing and immersion in citric acid, doxycycline, saline or chlorhexidine | Bacterial detoxification with acidic chemicals lowered osteoblastic cell viability and proliferation rates and induced changes in surface morphology and oxidation state of titanium, resulting in discoloration and pitting attack. | Biofilm-Treatment | (Ramesh et al., 2017) |
| Chemical | Carvacrol | Carvacrol significantly reduced biofilm formation compared to chlorhexidine against single- and multi-species biofilms. | Biofilm-Treatment and Biofilm-Prevention | (Ciandrini et al., 2014) |
| Electrochemical treatment | Low-current electrolysis | Electrolysis caused zero colony forming units at each of the anodic test discs and a reduction of total counts by three to five orders of magnitude at the cathode. | Biofilm-Treatment | (Sahrmann et al., 2014) |
| Electrochemical treatment | Electrochemical treatment with varying electrode potentials and electrolyte compositions | If the polarization potential was −0.5 or −1.0 V (vs. Ag/Ag+) the viable bacteria were reduced drastically though the disinfection was incomplete. The efficiency of the biofilm removal from titanium surfaces was significantly improved by decreasing the potential E b −1.4 V (vs. Ag/Ag+), by using a buffered solution with α-hydroxycarboxylic acids (mainly lactic acid) and by elongation of the electrolysis time. The final electrolysis parameters of 30 s at 7.0 V and 300 mA were sufficient to remove successfully 14-day old wildtype biofilms from dental implants *in-vitro*. | Biofilm-Treatment | (Schneider et al., 2018) |
| Peptide | DJK-5 and 1018 | A 6-h treatment by DJK-5 and 1018 (2 or 10 μg/mL) significantly reduced biomass of the multispecies biofilms on both Ti and HA disks. DJK-5 was able to kill more bacteria (40.4–75.9%) than 1018 (30.4–67.0%) on both surfaces. DJK-5 also led to a more effective killing of microbes after a 3-min treatment of 3-day-old and 3-week-old biofilms on Ti and HA surfaces, compared to peptide 1018 and chlorhexidine. No significant difference was found in the amount of biofilm treatment between Ti and HA surfaces. | Biofilm-Prevention | (Wang et al., 2018) |
| Peptide | GL13K | The GL13K coatings had a bactericidal effect and thus significantly reduced the number of viable bacteria compared to control surfaces. | Biofilm-Prevention | (Holmberg et al., 2013) |
| Peptide | hLf1-11 | Samples coated with the hLf1-11 peptide inhibited the early stages of bacterial growth. | Biofilm-Prevention | (Godoy-Gallardo et al., 2014) |
| Peptide | Mel4 and caspofungin | The peptides significantly inhibited the growth of *Candida albicans* and *Staphylococcus aureus*, thus inhibiting their ability to form biofilm on the surfaces. | Biofilm-Prevention | (Akhavan et al., 2018) |
| Peptide | Bifunctional chimeric peptides, comprising a combinatorially-selected titanium binding and computationally-designed antimicrobial domains (TiBPS-AMP) | The bacterial adhesion studies demonstrated that chimeric peptides coatings provided antimicrobial property for the titanium implants. | Biofilm-Prevention | (Yucesoy et al., 2015) |
| Probiotic | Live and heat-killed (HK) Lactic Acid Bacteria (LAB) | Live and HK *L. rhamnosus* ATCC 53103 and *L. paracasei* B21060 inhibited streptococci biofilm formation by competition and displacement mechanisms with no substantial differences. | Biofilm-Prevention | (Ciandrini et al., 2017) |

**References**

Akhavan, B., Michl, T. D., Giles, C., Ho, K., Martin, L., Sharifahmadian, O., et al. (2018). Plasma activated coatings with dual action against fungi and bacteria. *Appl. Mater. Today* 12, 72–84. doi:10.1016/j.apmt.2018.04.003.

Al-Hashedi, A. A., Laurenti, M., Amine Mezour, M., Basiri, T., Touazine, H., Jahazi, M., et al. (2018). Advanced inorganic nanocomposite for decontaminating titanium dental implants. *J. Biomed. Mater. Res. - Part B Appl. Biomater.*, 1–12. doi:10.1002/jbm.b.34170.

Baffone, W., Sorgente, G., Campana, R., Patrone, V., Sisti, D., and Falcioni, T. (2011). Comparative effect of chlorhexidine and some mouthrinses on bacterial biofilm formation on titanium surface. *Curr. Microbiol.* 62, 445–451. doi:10.1007/s00284-010-9727-x.

Bürgers, R., Witecy, C., Hahnel, S., and Gosau, M. (2012). The effect of various topical peri-implantitis antiseptics on Staphylococcus epidermidis, Candida albicans, and Streptococcus sanguinis. *Arch. Oral Biol.* 57, 940–947. doi:10.1016/j.archoralbio.2012.01.015.

Canullo, L., Genova, T., Wang, H.-L., Carossa, S., and Mussano, F. (2017). Plasma of Argon Increases Cell Attachment and Bacterial Decontamination on Different Implant Surfaces. *Int. J. Oral Maxillofac. Implants* 32, 1315–1323. doi:10.11607/jomi.5777.

Chen, C.-C. C.-J., Chen, C.-C. C.-J., and Ding, S.-J. (2016). Effectiveness of Hypochlorous Acid to Reduce the Biofilms on Titanium Alloy Surfaces in Vitro. *Int. J. Mol. Sci.* 17, 1161. doi:10.3390/ijms17071161.

Ciandrini, E., Campana, R., and Baffone, W. (2017). Live and heat-killed Lactobacillus spp. interfere with Streptococcus mutans and Streptococcus oralis during biofilm development on titanium surface. *Arch. Oral Biol.* 78, 48–57. doi:10.1016/j.archoralbio.2017.02.004.

Ciandrini, E., Campana, R., Federici, S., Manti, A., Battistelli, M., Falcieri, E., et al. (2014). In vitro activity of Carvacrol against titanium-adherent oral biofilms and planktonic cultures. *Clin. Oral Investig.* 18, 2001–2013. doi:10.1007/s00784-013-1179-9.

Cruz, H. V., Henriques, M., Teughels, W., Celis, J. P., and Rocha, L. A. (2015). Combined Influence of Fluoride and Biofilms on the Biotribocorrosion Behavior of Titanium Used for Dental Applications. *J. Bio- Tribo-Corrosion* 1. doi:10.1007/s40735-015-0021-0.

Diab Al-Radha, A. S., Younes, C., Diab, B. S., and Jenkinson, H. F. (2013). Essential Oils and Zirconia Dental Implant Materials. *Int. J. Oral Maxillofac. Implants* 28, 1497–1505. doi:10.11607/jomi.3142.

Dostie, S., Alkadi, L. T., Owen, G., Bi, J., Shen, Y., Haapasalo, M., et al. (2017). Chemotherapeutic decontamination of dental implants colonized by mature multispecies oral biofilm. *J. Clin. Periodontol.* 44, 403–409. doi:10.1111/jcpe.12699.

Fukushima, A., Mayanagi, G., Sasaki, K., and Takahashi, N. (2018). Corrosive effects of fluoride on titanium under artificial biofilm. *J. Prosthodont. Res.* 62, 104–109. doi:10.1016/j.jpor.2017.08.004.

Godoy-Gallardo, M., Mas-Moruno, C., Fernández-Calderón, M. C., Pérez-Giraldo, C., Manero, J. M., Albericio, F., et al. (2014). Covalent immobilization of hLf1-11 peptide on a titanium surface reduces bacterial adhesion and biofilm formation. *Acta Biomater.* 10, 3522–3534. doi:10.1016/j.actbio.2014.03.026.

Holmberg, K. V., Abdolhosseini, M., Li, Y., Chen, X., Gorr, S. U., and Aparicio, C. (2013). Bio-inspired stable antimicrobial peptide coatings for dental applications. *Acta Biomater.* 9, 8224–8231. doi:10.1016/j.actbio.2013.06.017.

Kotsakis, G. A., Lan, C., Barbosa, J., Lill, K., Chen, R., Rudney, J., et al. (2016). Antimicrobial Agents Used in the Treatment of Peri-Implantitis Alter the Physicochemistry and Cytocompatibility of Titanium Surfaces. *J. Periodontol.* 87, 809–819. doi:10.1902/jop.2016.150684.

Montelongo-Jauregui, D., Srinivasan, A., Ramasubramanian, A., and Lopez-Ribot, J. (2018). An In Vitro Model for Candida albicans–Streptococcus gordonii Biofilms on Titanium Surfaces. *J. Fungi* 4, 66. doi:10.3390/jof4020066.

Ntrouka, V., Hoogenkamp, M., Zaura, E., and Van der Weijden, F. (2011). The effect of chemotherapeutic agents on titanium-adherent biofilms. *Clin. Oral Implants Res.* 22, 1227–1234. doi:10.1111/j.1600-0501.2010.02085.x.

Ramesh, D., Sridhar, S., Siddiqui, D. A., Valderrama, P., and Rodrigues, D. C. (2017). Detoxification of Titanium Implant Surfaces: Evaluation of Surface Morphology and Bone-Forming Cell Compatibility. *J. Bio- Tribo-Corrosion* 3, 1–13. doi:10.1007/s40735-017-0111-2.

Roberts, J. L., Khan, S., Emanuel, C., Powell, L. C., Pritchard, M. F., Onsøyen, E., et al. (2013). An in vitro study of alginate oligomer therapies on oral biofilms. *J. Dent.* 41, 892–899. doi:10.1016/j.jdent.2013.07.011.

Sahrmann, P., Zehnder, M., Mohn, D., Meier, A., Imfeld, T., and Thurnheer, T. (2014). Effect of Low Direct Current on Anaerobic Multispecies Biofilm Adhering to a Titanium Implant Surface. *Clin. Implant Dent. Relat. Res.* 16, 552–556. doi:10.1111/cid.12018.

Schneider, S., Rudolph, M., Bause, V., and Terfort, A. (2018). Electrochemical removal of biofilms from titanium dental implant surfaces. *Bioelectrochemistry* 121, 84–94. doi:10.1016/j.bioelechem.2018.01.008.

Souza, J. G. S., Cordeiro, J. M., Lima, C. V., and Barão, V. A. R. (2018). Citric acid reduces oral biofilm and influences the electrochemical behavior of Titanium: An *in situ* and *in vitro* study. *J. Periodontol.* doi:10.1002/JPER.18-0178.

Verardi, G., Cenci, M. S., Maske, T. T., Webber, B., and Santos, L. R. dos (2016). Antiseptics and microcosm biofilm formation on titanium surfaces. *Braz. Oral Res.* 30, 1–6. doi:10.1590/1807-3107BOR-2016.vol30.0030.

Wang, D., Haapasalo, M., Gao, Y., Ma, J., and Shen, Y. (2018). Antibiofilm peptides against biofilms on titanium and hydroxyapatite surfaces. *Bioact. Mater.* 3, 418–425. doi:10.1016/j.bioactmat.2018.06.002.

Wiedmer, D., Petersen, F. C., Lönn-Stensrud, J., and Tiainen, H. (2017). Antibacterial effect of hydrogen peroxide-titanium dioxide suspensions in the decontamination of rough titanium surfaces. *Biofouling* 33, 451–459. doi:10.1080/08927014.2017.1322585.

Yucesoy, D. T., Hnilova, M., Boone, K., Arnold, P. M., Snead, M. L., and Tamerler, C. (2015). Chimeric Peptides as Implant Functionalization Agents for Titanium Alloy Implants with Antimicrobial Properties. *JOM* 67, 754–766. doi:10.1007/s11837-015-1350-7.
