# Supplementary figures and images for: Microbial Biofilm Decontamination on Dental Implant Surfaces: A Mini Review
Source: Front Cell Infect Microbiol. 2021 Oct 8;11:736186. doi: 10.3389/fcimb.2021.736186 (PMC8531646; doi:10.3389/fcimb.2021.736186)

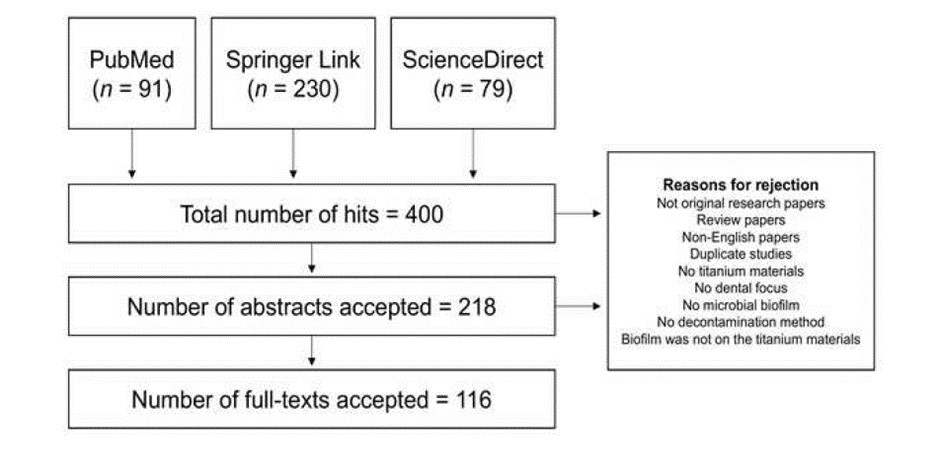

Supplement: Supplementary file 3 [file Image_1.jpg]
